# Corroles and Hexaphyrins: Synthesis and Application in Cancer Photodynamic Therapy

**DOI:** 10.3390/molecules25153450

**Published:** 2020-07-29

**Authors:** Susana M. M. Lopes, Marta Pineiro, Teresa M. V. D. Pinho e Melo

**Affiliations:** Coimbra Chemistry Centre and Department of Chemistry, University of Coimbra, 3004-535 Coimbra, Portugal; smlopes@uc.pt (S.M.M.L.); mpineiro@ui.uc.pt (M.P.)

**Keywords:** corroles, hexaphyrins, photodynamic therapy (PDT), photosensitizer dye

## Abstract

Corroles and hexaphyrins are porphyrinoids with great potential for diverse applications. Like porphyrins, many of their applications are based on their unique capability to interact with light, i.e., based on their photophysical properties. Corroles have intense absorptions in the low-energy region of the uv-vis, while hexaphyrins have the capability to absorb light in the near-infrared (NIR) region, presenting photophysical features which are complementary to those of porphyrins. Despite the increasing interest in corroles and hexaphyrins in recent years, the full potential of both classes of compounds, regarding biological applications, has been hampered by their challenging synthesis. Herein, recent developments in the synthesis of corroles and hexaphyrins are reviewed, highlighting their potential application in photodynamic therapy.

## 1. Introduction

**Figure 1:**
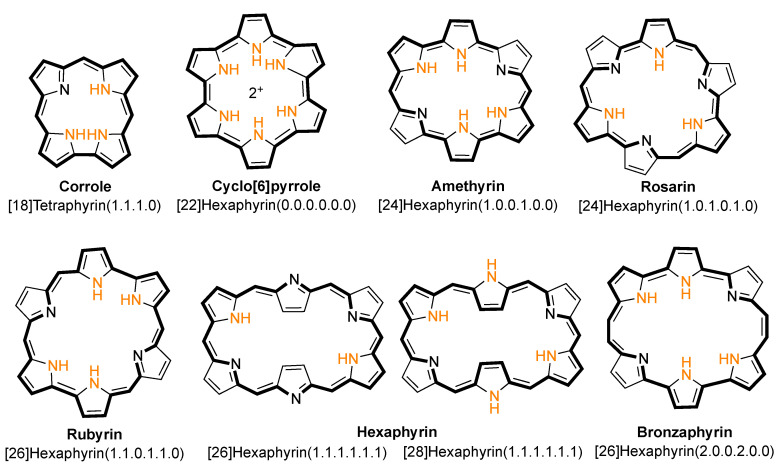
Chemical structures of corrole and hexaphyrins.

Corroles are tetrapyrrolic macrocycles containing 4 pyrrole units, 3 methine bridges and a direct pyrrole–pyrrole linkage (Figure 1). Therefore, corroles are contracted porphyrinoids with one methine (=CH–) bridge less than porphyrins, which leads to lower symmetry, higher fluorescence quantum yields, lower oxidation potentials and more intense absorption in the low-energy region of the visible spectrum. Despite being less studied than porphyrins over the years, probably due to their challenging synthesis, there was a significant change in this scenario after the synthetic breakthroughs by Gryko, Gross and Paolesse [1,2,3]. In fact, many efforts have been devoted to the synthesis of corroles in recent years [4,5,6,7]. Their free-bases and metal complexes have been explored in a wide range of applications [8,9], namely as promising drug candidates for prevention and treatment of diverse diseases, such as diabetes, neurodegenerative diseases and cancer [10,11,12,13,14], as antibacterial agents [15] as well as bio-imaging agents [16]. Corroles also found application in the photodynamic inactivation of microorganisms (PDI) [17], sonodynamic therapy [18] and photodynamic therapy (PDT) [19,20,21]. 

Expanded porphyrins [7,22,23,24] have gained remarkable attention due to their interesting and versatile features, such as diverse π-conjugation pathways owing to flexible structures [25], near- infrared (NIR) absorption/emission [26], facile interconversion between multiple redox states [27], and multi-metal coordination cavities for various divalent and trivalent metal ions (Figure 1) [22,28]. Beyond the study of these unique properties and their applications, the interest in expanded porphyrins has been focused on exploring the limits to which the classic Hückel definition of aromaticity may be applied. The goal has been to understand what factors endow a fully conjugated macrocycle with characteristics that can be considered aromatic or antiaromatic. Among the known expanded porphyrins that can combine from 5 to 12 pyrrole rings and diverse *meso*-bridges, hexaphyrins stand-out because these six-pyrrole macrocycles can adopt various conformations and electronic states such as Hückel aromatic, Hückel antiaromatic, Möbius aromatic, Möbius antiaromatic and stable radical states [29,30]. 

[26]Hexaphyrins(1.1.1.1.1.1) and [28]hexaphyrins(1.1.1.1.1.1), having six *meso*-bridges, are attractive molecules in view of aromaticity/antiaromaticity with a 26 and 28 π-electronic system, respectively, intense uv-vis absorption and a small HOMO-LUMO gap (Figure 1). [26]Hexaphyrins(1.1.1.1.1.1) can adopt a double-sided rectangular ring conformation with Hückel aromaticity while [28]hexaphyrins(1.1.1.1.1.1) adopt a single-sided twisted ring conformation with Möbius aromaticity. The unique uv-vis-NIR absorption/emission properties of these expanded π conjugated molecular systems offer useful optical nonlinear properties such as two-photon absorption (TPA) [31], which make them a promising class of two-photon absorption chromophores, with potential for a wide range of applications including microscopy, microfabrication, three-dimensional data-storage, optical power limiting, up-converted lasing, localized release of bio-active species, as well as application in photodynamic therapy [32].

## 2. Synthesis of New Corrole Derivatives

In recent decades, the synthesis of corroles and metallocorroles had attracted considerable attention from the scientific community and some reviews have been published [4,6,7,9,33,34]. Nevertheless, the relevance of corroles’ applications has driven the development of interesting approaches to construct macrocycles with new moieties, via classical and novel methodologies, collected herein.

### 2.1. Classical Synthetic Methodologies

One of the most explored routes to *meso*-substituted corroles relies on the synthesis of bilanes, obtained from the condensation of dipyrromethanes (DPs) with aldehydes, followed by oxidative macrocyclization. This strategy was applied to the synthesis of new corroles to be used in diverse applications.

**Scheme 1:**
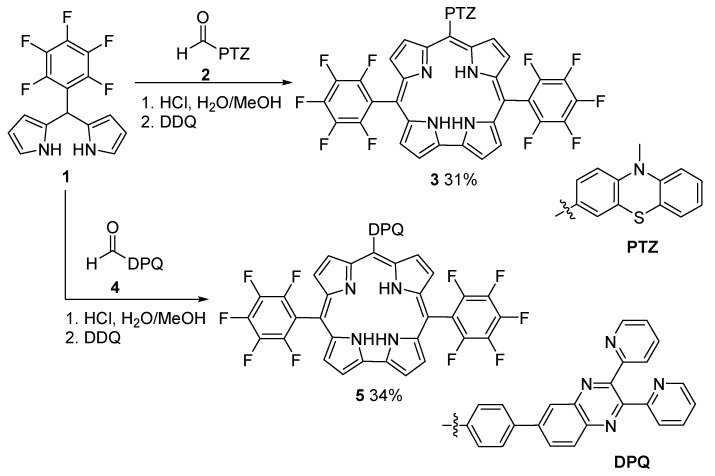
Synthesis of *trans*-A_2_B-corroles bearing phenothiazine and 2,3-di(2-pyridyl)quinoxaline substituents.

Liu and coworkers described the synthesis of *trans*-A_2_B corroles *meso*-substituted by phenothiazine (PTZ) and 2,3-di(2-pyridyl)quinoxaline (DPQ) groups. The HCl-catalyzed condensation of PTZ and DPQ formyl derivatives **2** and **4** with 5-pentafluorophenyldipyrromethane (**1**) followed by 2,3-dichloro-5,6-dicyano-1,4-benzoquinone (DDQ)-promoted macrocyclization gave the target corroles **3** and **5** in 31 and 34% yield, respectively (Scheme 1) [35]. Corroles **3** and **5** are novel donor-acceptor systems, in which the corrole scaffold is an acceptor and PTZ or DPQ moiety is an energy/electron donor. The absorption spectra of both dyads are a superposition of the absorption spectra of the monomers, indicating a weak electronic interaction between the corrole and either PTZ or DPQ groups, which was corroborated by the observed electrochemical properties. A detailed study of the excited state deactivation of both dyads shows that the singlet–singlet energy transfer from PTZ to the corrole unit is the main process in the case of dyad **3**, while in the case of **5** a reductive electron transfer, from the ground state of DPQ to the excited state of the corrole, is the main process. Therefore, one dyad mimics the primary process and the other mimics the reaction center events in photosynthesis. The proton-triggered emission studies of dyad **5** and OFET studies indicate that they can be applied in the DNA photocleavage.

**Scheme 2:**
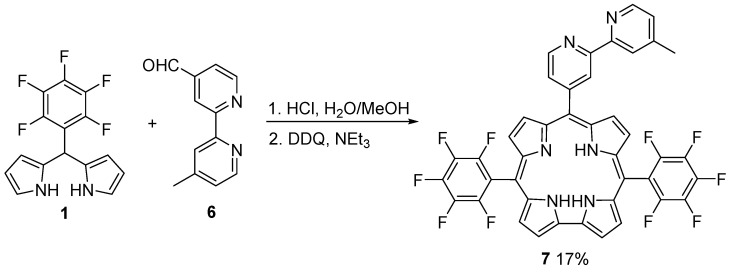
Synthesis of 10-(4-methyl-bipyridyl)-5,15-di(pentafluorophenyl)corrole.

The synthesis of 10-(4-methyl-bipyridyl)-5,15-di(pentafluoropheny)corrole (**7**) was achieved through Gryko’s methodology, i.e., the condensation of dipyrromethane **1** with the bipyridyl-aldehyde **6** in a mixture of H_2_O/MeOH using HCl as catalyst. Oxidation with DDQ afforded corrole **7** in 17% yield (Scheme 2) [36]. The uv-vis spectrum exhibits a strong Soret band at approximately 400 nm and the Q bands appear between 500 and 700 nm; a single emission band is observed in the fluorescence spectrum. The photophysical properties studied in dichloromethane (DCM) showed that corrole **7** fluorescence quantum yield is 0.04, the internal conversion quantum yield is 0.45 and the triplet quantum yield formation is about 0.54. The singlet oxygen quantum yield, a measure of the ability of the photosensitizer to generate cytotoxic singlet oxygen and an important parameter to evaluate the capability of the molecules to act as photosensitizers for PDT, was measured as 0.47, indicating a high efficiency in energy transfer from the corrole triplet state. 

**Scheme 3:**
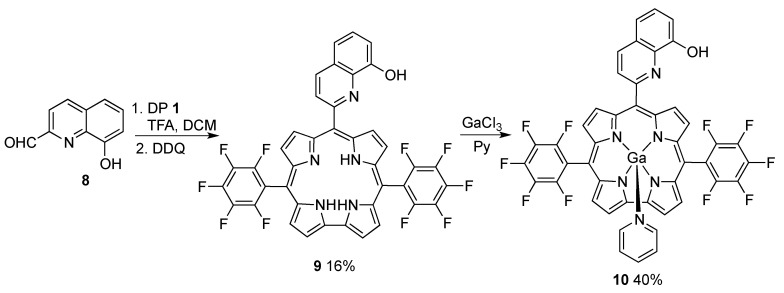
Synthesis of a 8-hydroxyquinoline-substituted A_2_B-type free base corrole and the corresponding Ga(III) corrole.

The trifluoroacetic acid (TFA)-catalyzed condensation of *meso*-pentafluorophenyl-dipyrromethane (**1**) with 8-hydroxyquilonine-2-carboxaldehyde (**8**) gave the low-symmetric A_2_B-type corrole **9** in 16% yield. Free base corrole **9** was treated with an excess of GaCl_3_ in refluxing pyridine to produce the Ga(III) corrole **10** in 40% yield (Scheme 3) [37]. According to the fluorescence studies, Ga(III) corrole **10** was a good “off-on-off” pH sensor in 1–14 pH range. The Ga(III) complex presents a fluorescence band with maximum at 603 nm, fluorescence quantum yield of 11.7% and a fluorescence lifetime of 2.42 ns in DCM solution. The intensity of fluorescence decreases gradually with the acidity of the solution and increases when the solution is more basic. 

**Scheme 4:**
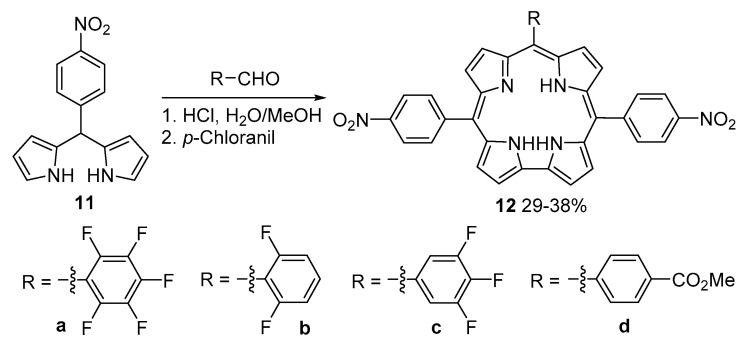
Synthesis of *trans*-A_2_B corroles [where A = 4-nitrophenyl and B = pentafluorophenyl, 2,6-difluorophenyl, 3,4,5-trifluorophenyl and 4-(methoxycarbonyl)phenyl groups].

The condensation of 5-(4-nitrophenyl)dipyrromethane (**11**) with aromatic aldehydes, followed by oxidation with *p*-chloranil allowed the isolation of *trans*-A_2_B corroles **12** in 29–38% yield (Scheme 4) [38]. The photophysical properties in toluene show characteristic absorption/emission spectra with Soret type bands between 420 and 450 nm, Q-type bands around 590–655 nm and the fluorescence bands in the range of 673–690 nm. The fluorinated corroles present fluorescence quantum yields higher than the carboxymethyl derivative, 0.76 for **12a**, 0.80 for **12b** and 0.77 for **12c**. Corroles **12a**–**d** showed an excellent affinity for the fluoride ions, allowing for an unusual selectivity for fluoride ions. The quenching of the fluorescence emission with the addition of fluorine ion indicates that these corroles can be used as fluorine ion sensors. 

**Scheme 5:**
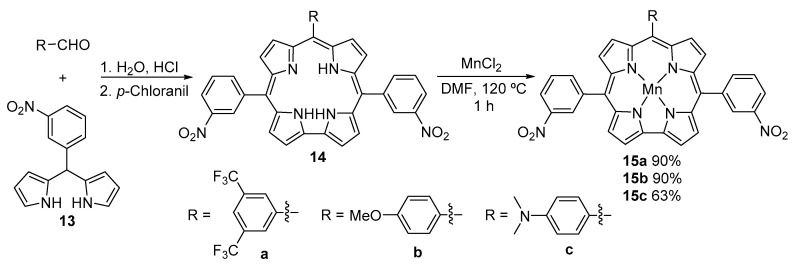
Synthesis of Mn(III) *trans*-A_2_B triarylcorroles [where A = 3-nitrophenyl and B = 4-methoxphenyl, 4-(dimethylamino)phenyl and 4-(trifluoromethyl)_2_phenyl groups].

Liang and coworkers developed the synthesis of three low-symmetry A_2_B type manganese(III) *meso*-triarylcorroles providing a push–pull electronic system (Scheme 5). The acid-catalyzed condensation of dipyrromethane **13** with the appropriate aldehyde followed by oxidation with *p*-chloranil gave corroles **14**. The reaction of these macrocycles with manganese chloride in DMF afforded metallocorroles **15a**–**c** in high yields (63–90%) [39]. The fluorescence studies reveal that Mn(III) corroles **15a**–**c** strongly interact with the cell-free circulating tumor DNA in solution and the ability of this interaction increases with the electron-donating character of the 10-substituent of the triarylcorrole. This interaction with DNA can be used for tumor detection and targeting drug delivery in vivo. 

**Scheme 6:**
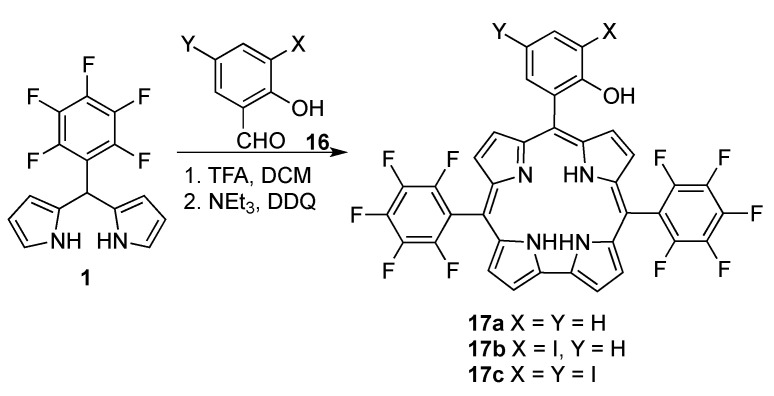
Iodine-containing A_2_B-type triaryl corroles synthesized to evaluate the influence of iodine atoms on the photophysical properties.

Triaryl corroles with a *meso*-iodoaryl substituent were prepared and their photophysical properties were studied in order to evaluate their capabilities as photosensitizers for PDT. A_2_B-type triaryl corroles **17** were synthesized through the TFA-catalyzed condensation of DP **1** with 2-hydroxybenzaldehyde and *m*-iodinated 2-hydroxybenzaldehydes, **16**, followed by oxidation with DDQ (Scheme 6) [40]. The presence of iodine atoms did not significantly influence the absorption spectra and only a slight decrease in the absorption coefficient of the Soret band (ca. 420 nm) was observed with the increase in the number of iodine atoms. However, the fluorescence quantum yield and fluorescence lifetime are strongly influenced by the iodine atom. Corrole **17a**, without iodine atoms, has a fluorescence quantum yield of 0.143 that decreases to 0.031 in the case of mono-iodo-corrole **17b** and to 0.023 for the di-iodo-corrole **17c**. The decrease in the fluorescence quantum yield is accompanied by the decrease in the fluorescence lifetime and a slight increase in the triplet quantum yields showcasing the heavy atom effect. Despite the increase in the efficiency of the triplet state formation, the singlet oxygen quantum yield (Φ_Δ_) decreased, going from mono-iodo-corrole **17b** (Φ_Δ_ = 0.9) to di-iodo-corrole **17c** (Φ_Δ_ = 0.4).

**Scheme 7:**
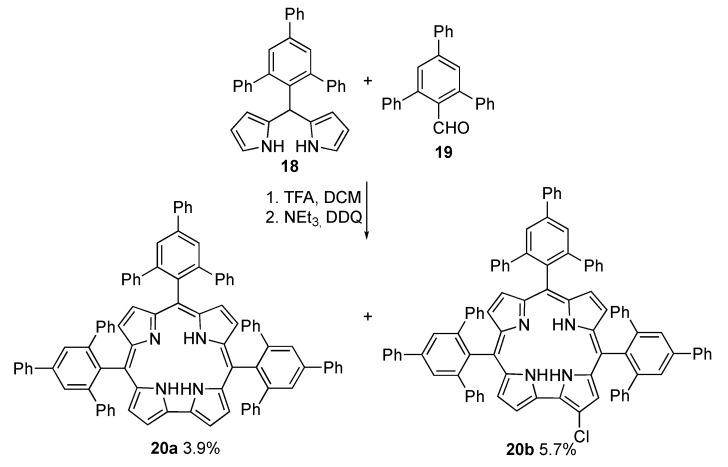
Synthesis of bulky bis-pocket corroles.

During the synthesis of the bulky bis-pocket corrole **20a**, Chang and coworkers identified another green compound as being the β-chlorinated corrole **20b**, fully supported by characterization data, including X-ray crystallography analysis (Scheme 7) [41]. The β-chloro-corrole **20b** was formed through the mono-chlorination of corrole **20a** in which DDQ acted as both oxidant and chlorinating agent. The presence of a chlorine atom at the β-position of corrole **20b** induced a slight red shift in the absorption and fluorescence emission spectra, together with a significant decrease in fluorescence intensity. Fluorescence lifetimes, as well as the fluorescence quantum yields, are shorter for β-chloro-corrole **20b** (2.5 ns) than for corrole **20a** (4.2 ns). However, the decrease in the fluorescence did not promote the increase in the capability to generate singlet oxygen, since non-chlorinated corrole **20a** demonstrated a higher ability to generate singlet oxygen than the β-chlorinated corrole **20b**.

**Scheme 8:**
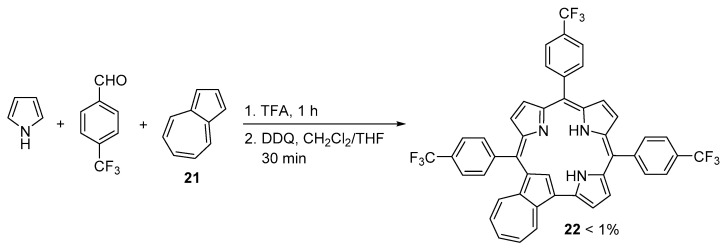
Synthesis of *meso*-tri-(*p*-trifluoromethyl)phenylazulicorrole.

The three component one-pot reaction of pyrrole, *p*-trifluoromethylbenzaldehyde and azulene (**21**), catalyzed by TFA, followed by oxidation with DDQ, allowed the isolation of novel *meso*-triarylazulicorrole **22** (Scheme 8) [42]. Despite the poor yield (<1%), carbacorrole **22** could be converted into the corresponding Cu(III) and Au(III) complexes. Single-crystal X-ray structures were obtained for the free-base and Cu(III) derivatives, uncovering detailed structural information on these novel macrocyles. The *meso*-triarylazulicorroles show absorption spectra with absorption features extending into the NIR region, opening the way to their application in bioimaging and photodynamic therapy. 

### 2.2. New Synthetic Methodologies

**Scheme 9:**
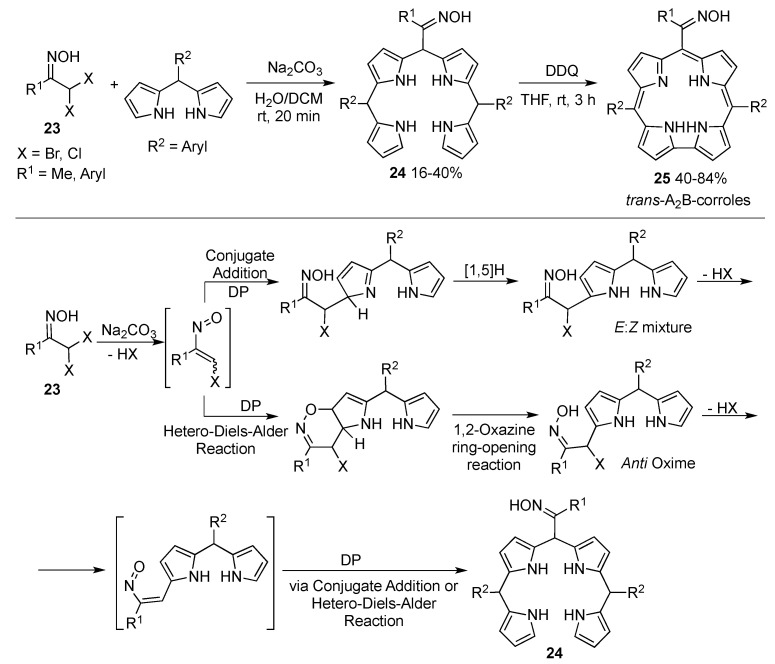
Synthesis of oxime-containing *trans*-A_2_B-corroles based on the reactivity of nitrosoalkenes toward dipyrromethanes.

The above-described syntheses of corroles were based on classical methodologies involving acid-catalyzed condensation of pyrroles or *meso*-substituted dipyrromethanes and aldehydes, followed by oxidation. Recently, Pinho e Melo et al. described an innovative synthesis of A_2_B-type corroles, bearing an oxime functionality, by exploring the reactivity of dipyrromethanes toward nitrosoalkenes (Scheme 9) [43]. In situ dehydrohalogenation of the α,α-dihalo-oximes **23** generates transient α-chloro-nitrosoalkenes, which react with dipyrromethanes either via hetero-Diels−Alder reaction or conjugated addition to give the corresponding alkylated dipyrromethanes. The side chains of these DPs undergo another dehydrohalogenation to afford new nitrosoalkenes, allowing the synthesis of the target bilanes **24** (16–40%) upon reaction with a second molecule of the dipyrromethane. The oxidative macrocyclization with DDQ affords the corresponding corroles **25** in 40–84% yields. These are the first examples of *meso*-substituted corroles with an oxime functionality.

## 3. Functionalization at the Inner Core and Peripheral Positions of Corroles

In addition to the synthesis of new corrole derivatives, the functionalization of the basic core of corroles is another aspect that has been largely used for the modulation of their properties. This includes the introduction of different metals and ligands in the internal cavity of the corrole or the introduction of functionalities, side chains or heterocycles, on the corrole’s periphery.

**Scheme 10:**
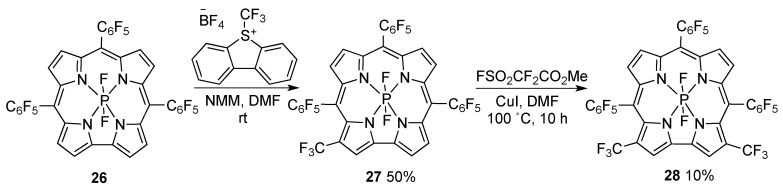
Introduction of CF_3_ substituents at β-positions of phosphorus(V) tri(pentafluorophenyl)corrole.

Gross and coworkers explored the influence of the presence of trifluoromethyl groups in the structure of phosphorus corroles as photocatalysts [44]. The authors developed several strategies to functionalize the phosphorus(V) tri(pentafluorophenyl)corrole (**26**) in order to obtain a library of complexes with different numbers of CF_3_ substitutions. Mixtures of monosubstituted corroles were obtained via direct electrophilic CF_3_ incorporation using FSO_2_CF_2_CO_2_Me and copper iodide. The mono-CF_3_-substituted corrole **27** could also be obtained in 50% yield carrying out the trifluoromethylation with 5-(trifluoromethyl)dibenzothiophenium tetrafluoroborate and *N*-methylmorpholine (NMM) (Scheme 10). The reaction of corrole **27** with FSO_2_CF_2_CO_2_Me/CuI afforded the bis-CF_3_-substituted derivative **28** in 10% yield. On the other hand, the synthesis of corroles with more than two CF_3_ groups required initial bromination leading to 2,3,8,17,18-pentabrominated derivatives, followed by treatment with the trifluoromethylating reagent (FSO_2_CF_2_CO_2_Me/CuI). The DFT analysis, electrochemistry and photophysical measurements reveal that compounds with a higher number of β-CF_3_ groups are the best photocatalysts for the bromination of phenol, toluene and benzene.

**Scheme 11:**
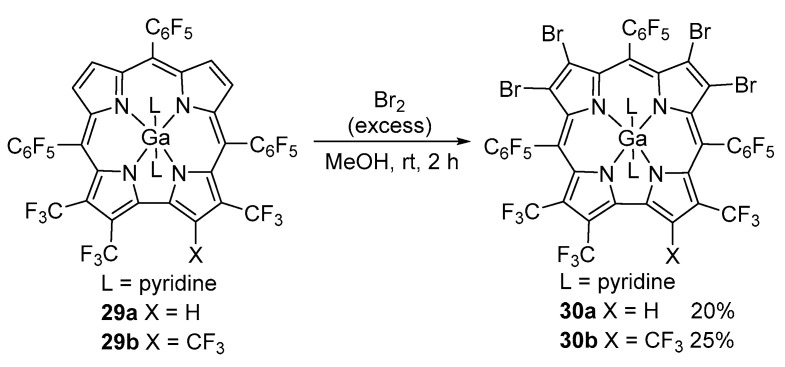
Tetra-bromination of tris- and tetra-CF_3_ substituted galium corrole complexes.

The same authors described the synthesis of tris- (**30a**) and tetra-CF_3_ substituted (**30b**) gallium corrole complexes bearing four bromine atoms at *beta*-positions (Scheme 11) [45]. A red shift was observed in the uv-vis absorption and emission spectra going from the non-brominated corroles to the tetra-brominated derivatives. Moreover, an increased reduction potential was observed, resulting in an enhancement performance of corroles **30**, especially corrole **30b**, regarding the photocatalytic bromide-to-bromine conversion when compared with octabrominated gallium corroles.

**Scheme 12:**
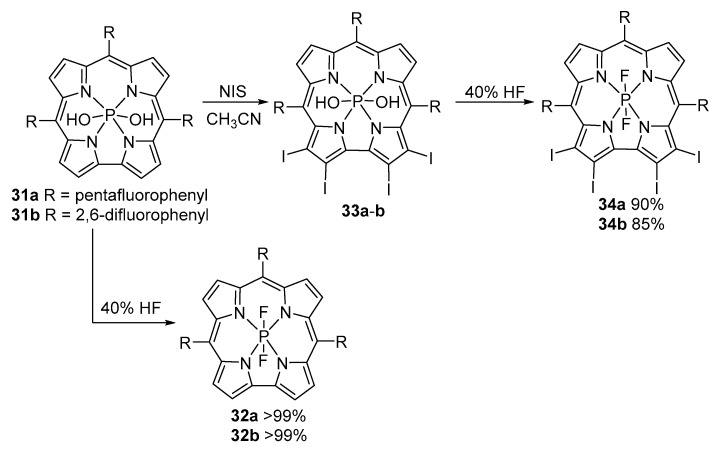
Axial ligand exchange and iodination of phosphorus corroles.

Gross and Zhao also described the synthesis and photophysical properties of fluorinated phosphorus corrole complexes **32** and the corresponding β-iodo-substituted derivatives **34 [46]**. The preparation of complexes **32** was achieved in quantitative yield by the axial hydroxyl ligand exchange of complexes **31** with fluorides, upon reaction with aqueous hydrofluoric acid (Scheme 12). The synthesis of corrole complexes **34** required the iodination of complexes **31** with *N*-iodosuccinimide (NIS) to give tetra-iodo corrole complexes **33**, whose subsequent treatment with aqueous hydrofluoric acid afforded the target molecules in high yields (85–90%). The presence of the iodine atoms red-shifts the absorption by 16–19 nm, and the absorption of the Q band (589 nm) is more intense. Corrole complexes **32** exhibit emission bands between 525 and 700 nm, while the iodine complexes **34** have emission bands at ca. 796 nm indicating that these emit phosphorescence. The fluorescence quantum yields and lifetimes decrease drastically with the addition of iodine atoms. On the other hand, the singlet oxygen quantum yields of the iodine-containing complexes **34** (60–71%) were higher than those observed for the non-iodinated complexes **32** (46–49%). These experimental results are in agreement with DFT and TDDFT computational calculations [47,48] performed with non-substituted and iodine substituted phosphorus corrole complexes. The results demonstrated that iodine-substituted phosphorus corrole complexes, such as **34**, are good candidates to act as photosensitizers in photodynamic therapy. 

**Scheme 13:**
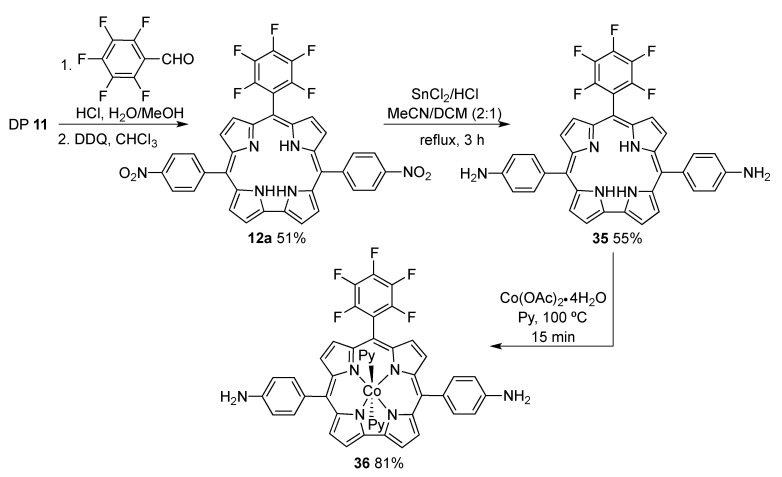
Synthesis of bis-aminophenyl-substituted Co(III) *trans*-A_2_B corrole (where A = *p*-aminophenyl and B = pentafluorophenyl).

Catalytic water splitting via oxygen evolution reaction (OER) and hydrogen evolution reaction (HER) are electrochemical processes that produce oxygen (O_2_) and hydrogen (H_2_), respectively, and constitute an interesting strategy for the conversion of renewable energy into chemical energy with applications in fuel cells and metal–air batteries. In this context, the bis-aminophenyl-substituted cobalt(III) corrole **36** was synthesized in order to evaluate its capability to act as an electrocatalyst for OER and HER (Scheme 13) [49]. Corrole **12a** was synthesized in high yield (51%) via HCl-catalyzed condensation of dipyrromethene **11** with pentafluorobenzaldehyde and converted into bis-aminophenyl-corrole **35** through the SnCl_2_/HCl reduction in the nitro groups. Reacting the free-base corrole **35** with Co(OAc)_2_·4H_2_O in refluxing pyridine, cobalt(III) corrole complex **36** was obtained in 81% yield. It was demonstrated that this aminophenyl-substituted cobalt(III) corrole acts as a bifunctional electrocatalyst for the oxygen and hydrogen evolution reactions. The strong electron-withdrawing pentafluorophenyl group present on the corrole ring was associated with the shift in the redox process towards the anodic direction and being responsible for the HER performance, whereas the increase in basicity due to the aminophenyl group was associated with the improvement in the OER activity.

**Scheme 14:**
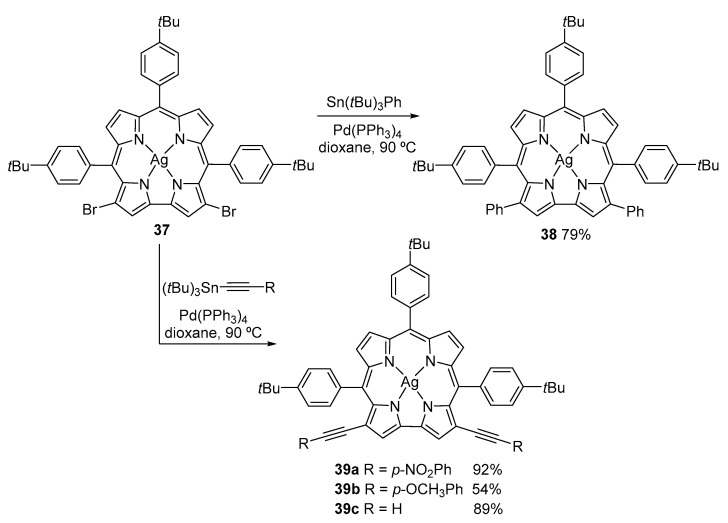
Synthesis of 3,17-diphenyl silver *meso*-arylcorrole complex and 3,17-diethynyl- 3,17-di(arylethynyl) silver *meso*-arylcorrole complexes.

The introduction of aryl and alkyl groups in the β-position of the *meso*-triarylcorrole complexes is a topic of interest, since it can lead to significant changes in their spectroscopic and electrochemical properties. Thus, Paolesse, Smith and coworkers applied the Stille reaction to introduce aryl and ethynyl groups at β-positions of silver corrole complex **37** [50]. Thus, the reaction of corrole **37** with tributylphenyl tin or tributylethynyl tin derivatives in presence of palladium tetrakis(triphenylphosphine) gave β-aryl silver corrole complex **38** in high yields, as well as 3,17-diethynyl-substituted and 3,17-di(arylethynyl)-substituted silver corrole complexes **39** (54–92%) (Scheme 14). The introduction of acetylenic moieties is particularly interesting since it opens the possibility to explore “click” reactions as a route to corrole-based materials/conjugates. β-Ethynyl/arylethynyl silver corrole complexes **39** exhibit red shifts (ca. 20 nm) of the B and Q bands in the absorption spectra when compared with the corresponding unsubstituted or halogenated silver corroles. DFT calculations can assign the clear impact, especially on the Q bands, of the conjugation of the β-ethynyl/arylethynyl units with the corrole π-system, made possible by the coplanar orientation of the acetylenic groups at the beta-positions with the corrole macrocycle. The shift in the B and Q bands towards lower energies was more pronounced for *p*-nitrophenylethynyl derivative **39a**. This increase in the absorption within the therapeutic window (650–800 nm) points to the applicability of this compound in photodynamic therapy.

**Scheme 15:**
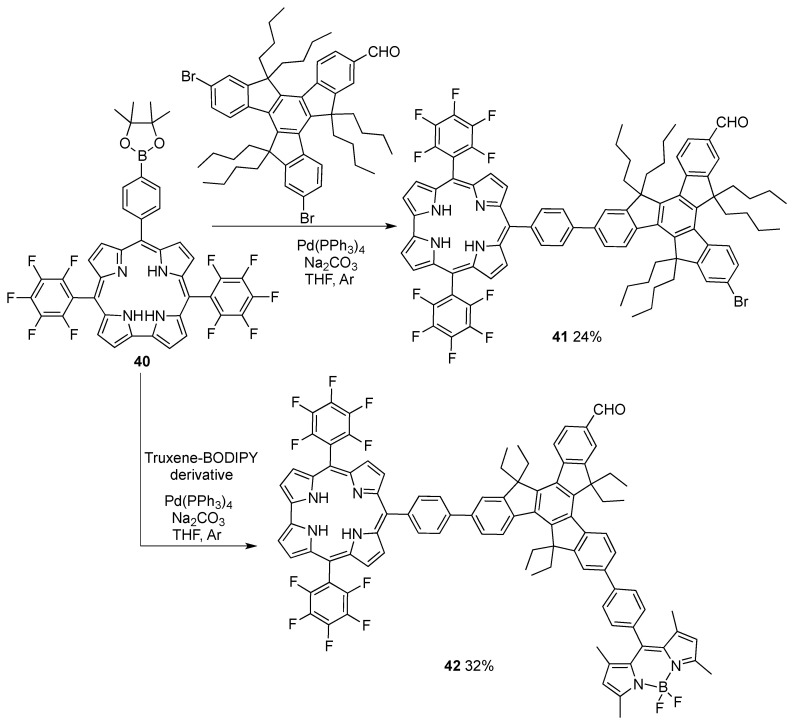
Synthesis of a truxene-corrole derivative and a truxene-bridged BODIPY-corrole dyad.

Truxene-corrole **41** and the truxene-bridged BODIPY-corrole dyad **42** were obtained from the reaction of corrole **40** with a truxene derivative or with the truxene-BODIPY derivative, as outlined in Scheme 15 [51]. Truxene-bridged BODIPY-corrole dyad **42** shows strong and broad absorption between 300 and 676 nm, i.e., the combination of the BODIPY and corrole chromophores allowed to improve the uv-vis absorption characteristics of the corrole core, with their use as photosensitizers in mind. The dyad showed fast singlet–singlet energy transfer from BODIPY to corrole, resulting in a triplet state with characteristics (energy and lifetime) that are more adequate to achieve an efficient energy transfer to oxygen, which results in a higher singlet oxygen photosensitizing ability (70% for **42**) when compared with the corresponding corrole (58%). Moreover, when the dyad is used as triplet photosensitizer for triplet−triplet annihilation upconversion, with red excitation at 589 nm, the upconversion emission intensity is higher than the corrole itself and the upconversion quantum yield (2.57%) increases by ca. 2-fold. The combination of BODIPY and corrole results in a dyad with improved photophysical properties to be used as a photosensitizer for PDT.

**Scheme 16:**
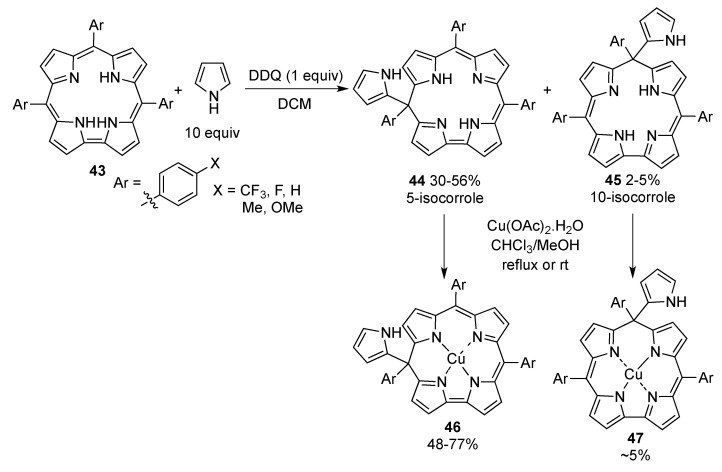
Synthesis of pyrrole-appended *meso*-triarylisocorroles and the corresponding copper(II) complexes.

Ghosh and coworkers described the synthesis of *meso*-pyrrole–appended isocorroles via oxidative coupling of corroles with pyrrole. The treatment of triarylcorroles **43** with an excess of pyrrole in the presence of DDQ gave 5-isocorroles **44** and 10-isocorroles **45** (Scheme 16) [52]. 5- Isocorroles **44** were isolated in higher yields (30–56%) than the isomeric 10-isocorroles **45** (2–5%). The pyrrole-appended isocorroles could be further complexed with Cu(II) in moderate yield by reacting with copper(II) acetate. Single-crystal X-ray structures were reported for two of these interesting macrocycles. The uv-vis spectra of free base isocorroles (**44** and **45**) and the corresponding Cu(II)-complexes (**46** and **47**) show absorption in the NIR region. The Cu(II) complexes exhibit more significant red-shifted spectra, relatively, to the free bases for both Soret and Q bands. The most significant shift was observed for the strong Q bands, which appear broad and double-humped at about 650–725 nm in the case of the free bases, whereas the Cu complexes also exhibit double-humped Q bands but sharper and red-shifted bands into the near-infrared (800–855 nm). These features make the free base isocorroles and their Cu complexes potential agents for biological applications, namely, as photodynamic agents.

**Scheme 17:**
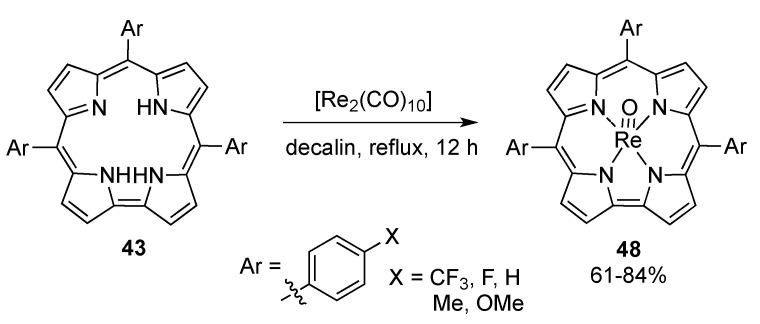
Synthesis of Re(V)-oxo triarylcorroles.

Ghosh and coworkers also described the efficient synthesis, single-crystal X-ray structure analyses and photophysical properties of rhenium(V)-oxo triarylcorroles **48** (Scheme 17). Macrocycles **48** were isolated in high yields (61–84%) by oxidative metalation of *meso*-triarylcorroles **43** with dirhenium decacarbonyl in refluxing decalin (Scheme 17) [53]. The different metallo-triarylcorrole derivatives **48** exhibit very similar uv-vis spectra despite bearing different *meso*-aryl-substituents. Rhenium complexes **48** show moderate near-infrared phosphorescence (from λ_em_ = 770 to λ_em_ = 788 nm) with lifetimes between 56 and 74 μs and quantum yields ranging from 1.07 to 1.52%. The dyes were evaluated as potential optical oxygen sensors and as sensitizers in triplet–triplet-annihilation-based upconversion. It was demonstrated that ReO corroles are viable sensitizers for triplet–triplet-annihilation-based upconversion, covering the orange part of the electromagnetic spectrum (λ_abs_ = 590 nm and upconversion quantum yield = 0.16). These features and the excellent photostability of the rhenium(V)-oxo corroles **48** make them good candidates to be used as sensitizers in photodynamic therapy [54].

**Scheme 18:**
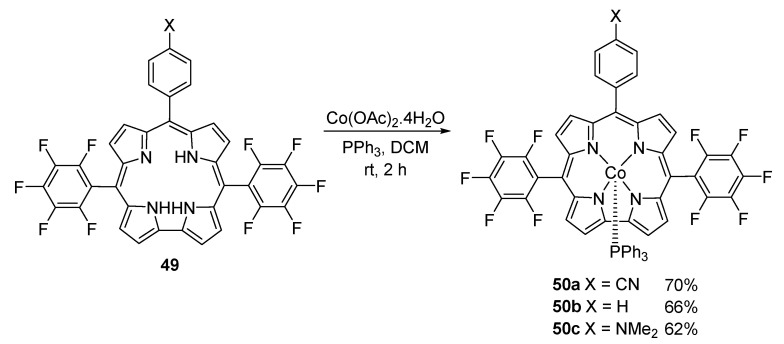
Synthesis of Co(III) *trans*-A_2_B triarylcorrole complexes (where A = pentafluorophenyl and B = phenyl or *p*-substitutedphenyl).

Cobalt(III) corroles **50**, substituted at the 10-*meso*-phenyl position by an electron-withdrawing or electron-donating group, were prepared in high yields (62–70%) by metalation of the corresponding free base corroles **49** with cobalt acetate in the presence of triphenylphosphine (Scheme 18) [55]. These corroles act as electrocatalysts in hydrogen evolution reactions. Showing high stability under electrolysis conditions, complex **50a** was more active than **50b** and **50c**. This suggests that the introduction of suitable peripheral substituents is a way to modulate the catalytic performance of Co(III) corroles.

**Scheme 19:**
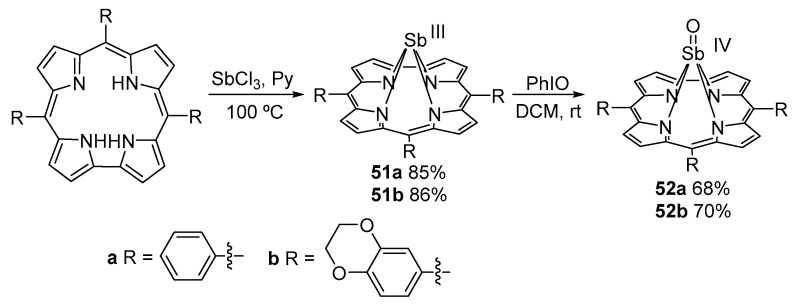
Synthesis of corrole antimony(III) complexes via metalation of triarylcorroles and their oxidation into corrole (oxo)antimony(V) complexes.

Aiming to develop photocatalysts, the synthesis of antimony(III) corrole complexes **51** and (oxo)antimony(IV) corrole complexes **52** was described by the group of Kar [56]. The Sb(III) complexes **51** were obtained in good yields (85–86%) by the metalation of the corresponding free base corroles with antimony(III) chloride in pyridine. The oxidation with iodoxobenzene in dichloromethane gave the (oxo)antimony(IV) complexes **52** (Scheme 19). The uv-vis spectra of complexes **51** show split Soret bands at 440–462 nm, while complexes **52** exhibit a single Soret band at 408–413 nm, which indicates that complexes **51** are less symmetric than **52**. Corrole complexes **51** and **52** show weak emission at 630–649 nm and a shoulder at 688–711 nm. 

**Scheme 20:**
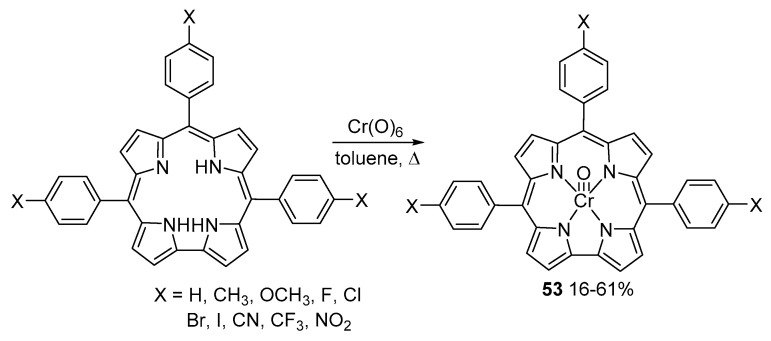
Synthesis of Cr(V) *meso*-triarylcorrole complexes.

The A_3_-type chromyl(V) triarylcorrole complexes **53** were prepared from the corresponding free base corroles in moderate yields by metalation with chromium oxide in refluxing toluene (Scheme 20) [57]. Even though having high similarity in electronic and structural properties, complexes **53** show some differences in redox potentials, which were associated with the inductive effect of the substituents at the 4 position of the aryl groups.

**Scheme 21:**
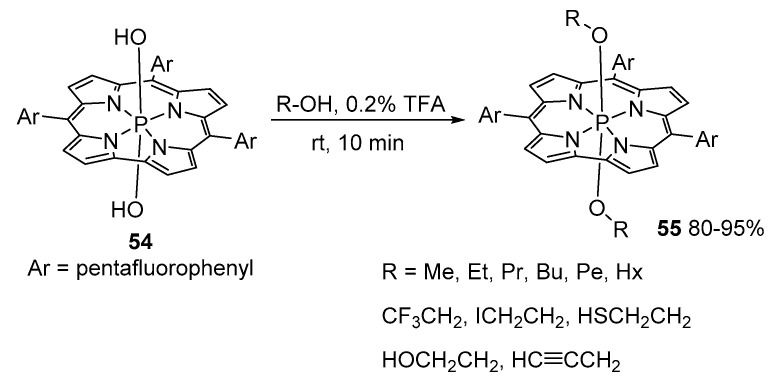
Acid-catalyzed axial ligand exchange in phosphorus corrole complexes.

Axial ligand exchange is another way to modulate the properties of phosphorus corrole complexes. The treatment of corrole dihydroxophosphorus (V) complex **54** with the appropriate alcohol in presence of TFA, at room temperature during 10 min, afforded corrole complexes **55** in high yields (80–95%) (Scheme 21) [58]. This acid-catalyzed ligand exchange was followed by uv-vis and ^31^P-NMR spectroscopy analyses. The absorption and emission spectra of corrole complexes **54** and **55** were very similar. Fluorescence quantum yields for complexes **55** (28–30%) are slightly lower than the ones observed for complex **54** (32%). This decrease was accompanied with shorter fluorescence lifetimes. The study of six-coordinate phosphorus corroles included derivatives with axial ligands containing terminal halides, thiols and alkenyl groups, which are very interesting functionalities since they open the way for further functionalization in order to enhance photophysical properties and applicability.

**Scheme 22:**
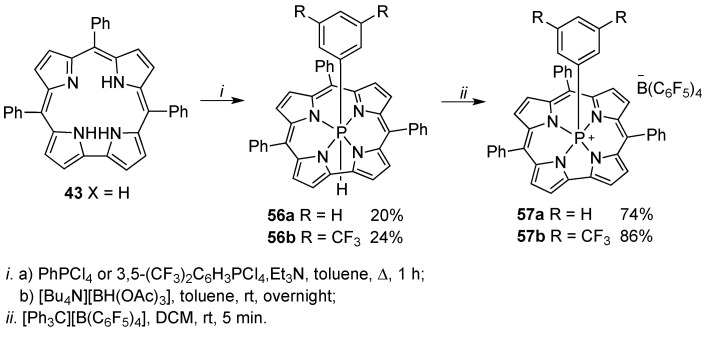
Synthesis of tetragonal phosporus(V) cations.

A series of tetragonal phosphorus(V) cations (e.g., **57**) have been prepared, characterized and their ability to act as Lewis acid catalysts in selected chemical transformations evaluated (Scheme 22) [59]. The cationic phosphoracorroles **57** were prepared in two steps, starting from the free base *meso*-triphenylcorrole. Treatment of corrole **43** with phenyl tetrachlorophosphorane or 3,5-bis(trifluoromethyl)phenyl tetrachlorophosphane in presence of trimethylamine, followed by addition of tetrabutylammonium triacetoxyborohydride, afforded the hexacoordinate corroles **56a** and **56b**, respectively. The second step was the treatment of corroles **56** with [Ph_3_C][B(C_6_F_5_)_4_] which led to tetragonal phosphorus(V) cations **57**. These cationic phosphoracorroles demonstrated high tolerance toward hydroxylic functionalities and proved to be very efficient Lewis acid catalysts for carbonyl hydrosilylation, C_sp3_–H bond functionalization and carbohydrate deoxygenation reactions.

**Scheme 23:**
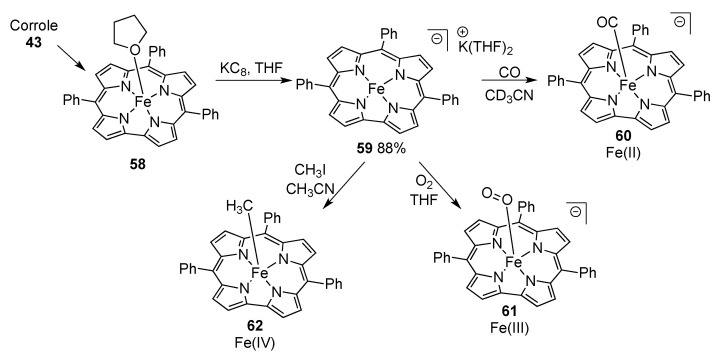
Synthesis of iron corrole anions.

The synthesis of iron(II) corrolate **59** has been described, as well as its reactivity towards molecular oxygen and organic electrophiles (Scheme 23) [60]. The reaction of corrole **58** with stoichiometric quantities of KC8 in tetrahydrofuran afforded the iron(II) corrolate **59**, K(THF)2[Fe(TPC)]. This corrole complex of divalent iron could be isolated and crystallographically characterized. Corrole **59** reacts with carbon monoxide to generate the anion **60**, with molecular oxygen, giving the Fe(III) corrole **61**, and with methyl iodine, affording the Fe(IV)-methyl complex **62**.

**Scheme 24:**
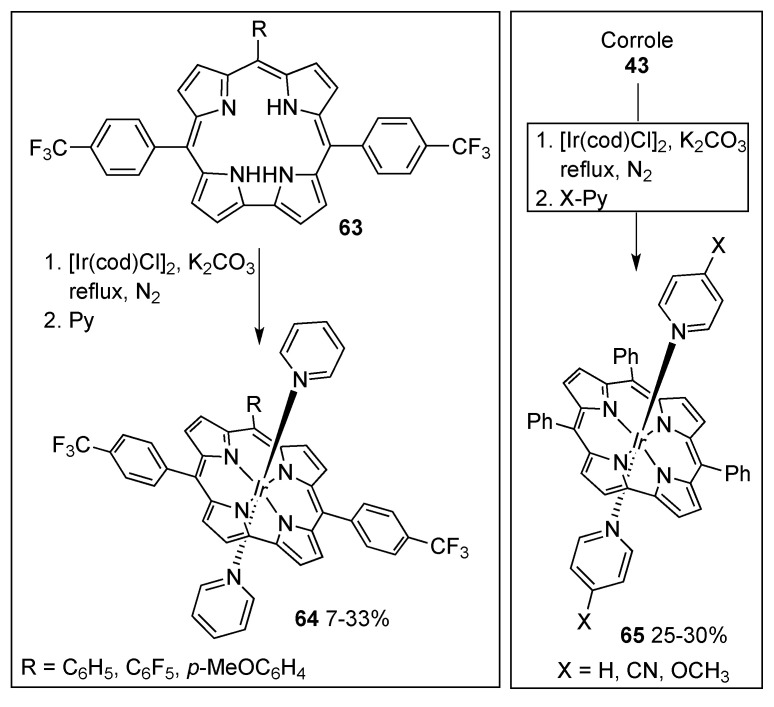
Synthesis of A_2_B type Ir(III) triarylcorroles and A_3_ type Ir(III) triarylcorroles.

Liang and coworkers described the synthesis and the optical and redox properties of A_2_B type Ir(III) triaryl corroles **64** containing *meso*-substituents with push–pull effect and A_3_ type triphenyl corroles **65**, differing in the axial pyridine ligands (Scheme 24) [61]. The metalation of the A_2_B type corroles **63** leading to iridium(III) triaryl corroles **64** was carried out upon reaction with [Ir(cod)Cl]_2_ in the presence of potassium carbonate in refluxing THF, followed by the addition of pyridine. The A_3_ type Ir(III) triphenyl corroles **65** were prepared by a similar metalation procedure starting from the triphenyl corrole **43**, using *p*-substituted pyridines in the last step. The Ir(III) corroles **64** and **65** demonstrated similar optical and redox properties, showing interesting electrocatalytic properties in oxygen reduction reactions. The nature of the *meso*-substituents of the A_2_B type corroles and the nature of the axial pyridine ligands of the A_3_ type Ir(III) triarylcorroles modulates their reactivity. 

Photophysical studies of six-coordinated Ir(III) triarylcorroles showed that regardless of the aryl substituent (phenyl, *p*-methoxyphenyl, *p*-methylphenyl, *p*-fluorophenyl or *p*-trifluoromethylphenyl) and axial ligand (pyridine, trimethylamine, isoquinoline, *p*-dimethylaminopyridine or *p*-picolinic acid) these complex exhibit low phosphorescence at room temperature. Remarkably, Ir(III) complex of 5,10,15-tris(*p*-trifluorophenyl)corrole were found to be singlet oxygen sensitizers. The ability to produce singlet oxygen was found to be dependent on the axial coordination. The complex bearing *p*-dimethylaminopyridine as an axial ligand presents a singlet oxygen formation quantum yield of 0.38, while the complex with pyridine as an axial ligand shows a significant increase in singlet oxygen formation quantum yield to 0.71. Thus, iridium corroles show adequate characteristics to be used as PDT sensitizers [62].

**Scheme 25:**
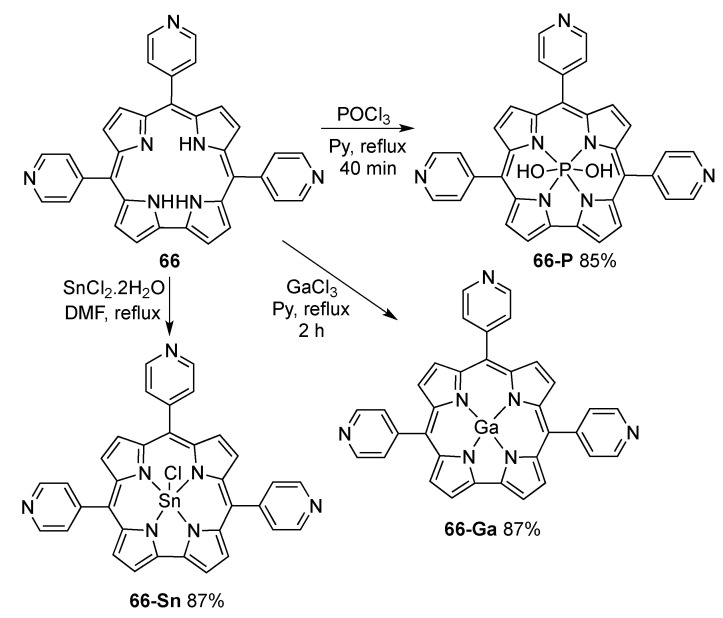
Synthesis of phosphorus, gallium and tin complexes of *meso*-tri(4-pyridyl)corrole.

Phosphorus (**66**-**P**), gallium (**66**-**Ga**) and tin (**66**-**Sn**) complexes were prepared in high yields (85–87%) by the reaction of tri(4-pyridyl)corrole **66** with POCl_3_ or the appropriate metal source (Scheme 25) [63]. These corrole derivatives exhibit sharper and stronger Soret bands that the free base corrole **66**, the major red-shift being observed for corroles **66**-**Sn** and **66**-**Ga**. High fluorescence quantum yields were found for complexes **66**-**P** (0.41) and **66**-**Ga** (0.31) while complex **66**-**Sn** has a fluorescence quantum yield lower than that observed for free base corrole **66** (0.12). Complex **66**-**Sn** has the lowest fluorescence lifetime (0.2 ns) and free base corrole **66** the highest (3.2 ns), whereas complexes **66**-**P** and **66**-**Ga** have similar fluorescence decays, 2.3 and 2.1 ns, respectively. Complex **66**-**Sn** has the highest singlet oxygen quantum yield (0.71) which, together with its great photostability, makes it an interesting photosensitizer. 

## 4. New Materials Containing Corroles

In recent years, new materials containing the corrole motif, such as nanoparticles, metal organic frameworks (MOFs), covalent organic framework (COF) and porous organic polymers (POPs), have attracted the interest of the scientific community.

**Scheme 26:**
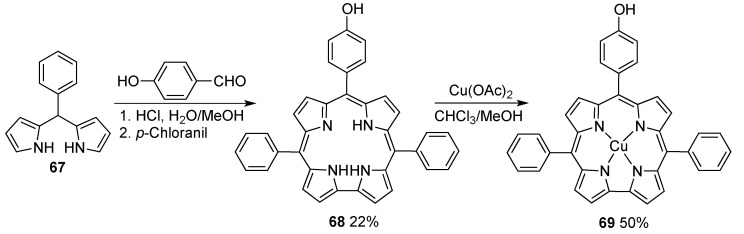
Synthesis of *trans*-A_2_B corrole copper complex (where A = phenyl and B = *p*-hydroxyphenyl).

Lu and coworkers described the synthesis of 10-(*p*-hydroxyphenyl)corrole copper complex **69** and its corresponding self-assembled nanostructure [64]. The free base corrole **68** was prepared in 22% yield by condensation of 5-phenyldipyrromethane (**67**) and *p*-hydroxybenzaldehyde in the presence of HCl, followed by oxidation with *p*-chloranil (Scheme 26). Metalation of the free base corrole **68** with copper(II) acetate gave the expected complex **69** in good yield (50%). The copper corrole **69** self-assembled into large-scale, needle-like nanostructures, with H-aggregate nature and relevant intermolecular π-π interactions. These nanostructures revealed high electric conductivity, acting as gas sensors with high selectivity for NO_2_ gas. 

**Figure 2:**
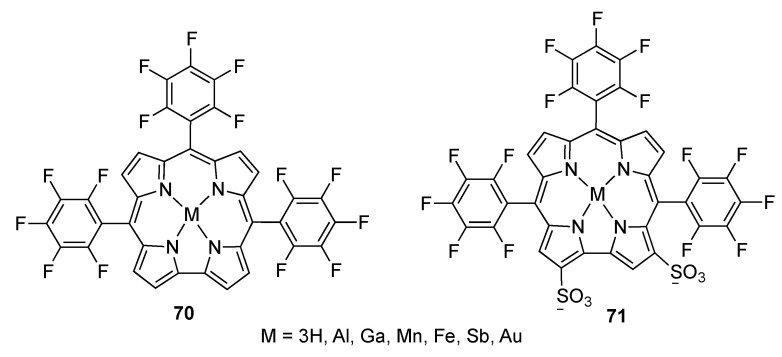
Structure of metallo *meso*-tri(pentafluorophenyl)corroles.

Contrary to porphyrins, where aggregates have been deeply investigated, the aggregation of corroles has been less explored. Nevertheless, it has been demonstrated that in aqueous media and in biological conditions, hydrophobic corroles and metallocorroles form nanoparticles (NPs) due to aggregation processes. In fact, stable nanoparticles were prepared by rapid injection of DMSO or pyridine solutions of tris-pentafluorophenyl corrole complexes **70** (M = Ga, Al and Au) in aqueous media (Figure 2). The analysis of uv-vis, IR spectra and DFT calculations of these NPs showed only small changes in comparison to the corresponding monomer, which was attributed to very weak intermolecular interactions in these nanoparticles [65].

Gross and coworkers studied the correlation between the lipophilicity/hydrophilicity of corroles and their metal complexes and their capability to act as anticancer agents. The authors found high affinity of corroles with the very-low-density lipoprotein fraction (VLDL) of human serum and high photocytotoxicity [66]. It was observed that the cytotoxicities increased in the order Ga < Fe < Al < Mn < Sb < Au for the studied corroles [67]. They studied assemblies of NPs of corroles with native serum proteins, soluble in aqueous media, to act as controlled release drug delivery systems. The formulation of NPs using specific proteins enables the targeting cells in which the corresponding receptors are overexpressed, and therefore increases the selectivity of corroles for the targeted tissue. The cellular uptake in DU-145 prostate cancer cells, followed by optical imaging, reveals that transferrin/corrole **70** nanoparticles were internalized, with distribution into the endoplasmic reticulum and lysosomes, to a greater extent by the transferrin-receptor-rich DU-145 cells, than albumin/corrole **70** NPs. Moreover, the stability and enhanced uptake properties of transferrin/corrole **70** NPs indicate that these can be applied as imaging agents (Figure 2) [67].

**Scheme 27:**
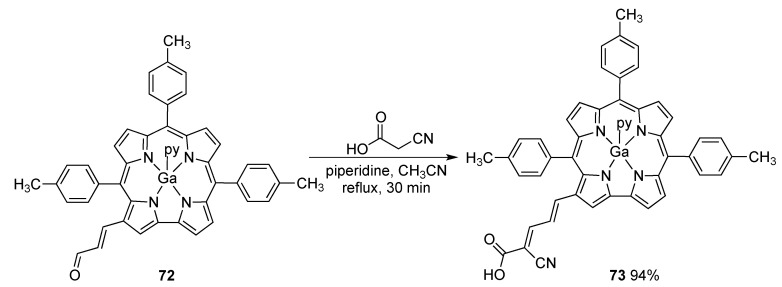
Knoevenagel reaction to introduce a monodentate anchoring group into an acrolein-substituted gallium corrole complex.

Gallium corrole-coated zinc oxide (ZnO) nanoparticles were prepared by the addition of corrole **73** to an ethanol solution of zinc acetate (nanoparticle precursor). Knoevenagel reaction with cyanoacetic acid was used to introduce a monodentate anchoring group into acrolein-substituted gallium corrole complex **72**, leading to the target corrole **73** in high yield (94%) (Scheme 27) [68]. The presence of the α-cyanocarboxylic group affects the photophysical properties of the corrole **73**, namely, a red-shift was observed in the Soret and Q bands, the fluorescence emission was also redshifted (32 nm) and the fluorescence quantum yield decreased to 2.2%. The nanoparticles showed a diameter smaller than 100 nm and the presence of the corrole macrocycle in the hybrid material could be confirmed by fluorescence microscopy analysis. The proprieties of the hybrid nanostructured material incorporating gallium corrole dyes indicates a large range of potential applications, namely, their use in medical diagnostic or chemical sensors fields.

**Scheme 28:**
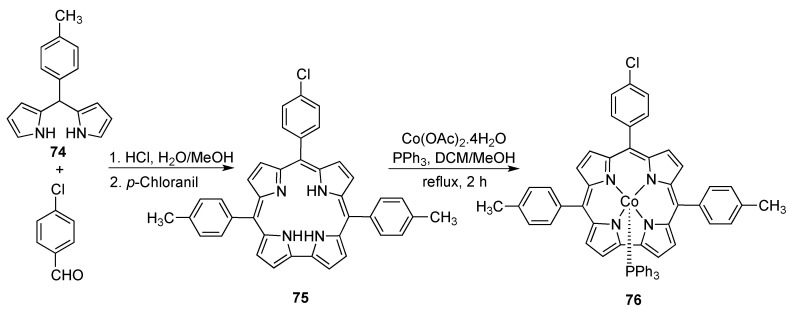
Synthesis of *trans*-A_2_B 10-(4-chlorophenyl)-5,15-di(4-methylphenyl)-corrole and its cobalt(III) complex.

Tang and coworkers synthesized *trans*-A_2_B *meso*-(4-chlorophenyl)-corrole **75** and the corresponding cobalt(III) complex **76** (Scheme 28) which was incorporated onto graphene oxide (GO) through π-π interactions. The gas sensing ability of corroles **75**, **76** and the corrole-functionalized GO was evaluated. Cobalt(III) complex **76** exhibits better NO_2_ gas sensitivity than the corresponding free-base corrole (**75**), due to the presence of the coordination metal. Likewise, the Co(III)corrole complex functionalized GO also demonstrated higher gas sensitivity [69].

**Scheme 29:**
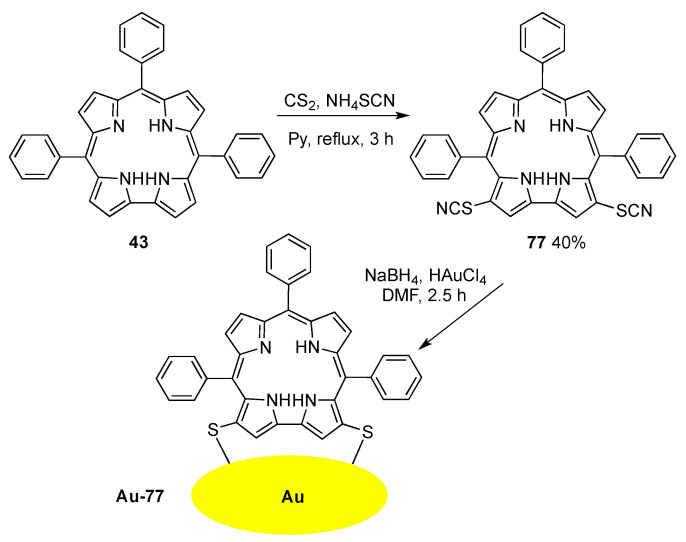
Thiocyanation of *meso*-triphenylcorrole and schematic representation of gold-corrole nano-assemblies.

A simple method for the regioselective thiocyanation of 5,10,15-triphenylcorrole (**43**) was developed by Kar’s group. The treatment of a solution of corrole **43** in carbon disulfide with an excess of ammonium thiocyanate (NH_4_SCN) at reflux, gave the β-thiocyanate corrole **77** in 40% yield (Scheme 29) [70]. Gold nanoparticles coated with the β-thiocyanate corrole, **Au**-**77**, were prepared by the addition of sodium borohydride to a premixed mixture of corrole and tetrachloroauric acid (HAuCl_4_). The formation of **Au**-**77** was monitored by uv-vis spectroscopy, starting from a solution of corrole **77** in DMF, with no significant changes upon addition of NaBH_4_ but both the Soret and Q bands of corrole underwent drastic changes in intensity upon the addition of the HAuCl_4_ solution. The final absorption spectra of **Au**-**77** show the presence of the characteristic Soret band from the corrole **77**, a strong plasmon resonance band of gold nanoparticles (525 nm) and the presence of Q bands (655 nm), despite being significantly reduced. The intensity of emission bands is significantly reduced, going from corrole **77** to **Au**-**77**, the same trend observed for the fluorescence lifetime, from 3.69 to 1.19 ns, respectively. A plausible structure of gold-corrole nano-assemblies was proposed based on a face-off binding mode of corrole, which would be possible due to pyrrole–pyrrole linkage (lack of one *meso*-carbon). The **Au**-**77** nanoparticle size is about 6.5 nm and they are stable under ambient conditions, which makes the corrole framework an excellent stabilizer for gold nanoparticles. 

**Scheme 30:**
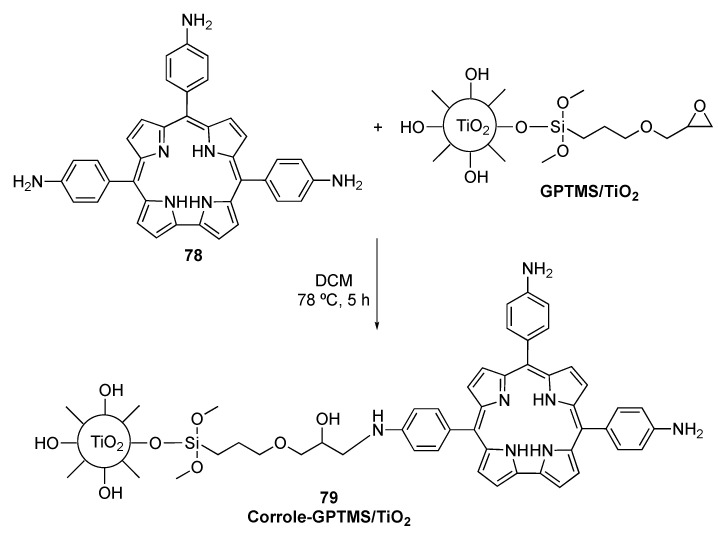
Design of the photocatalyst corrole-GPTMS/TiO_2_ nanoparticles.

The synthesis of an organic–inorganic photocatalyst to promote the photodegration of the pollutant di-anionic azo-dye Acid Black 1 was described by Zhu and coworkers. This photocatalyst consists of corrole **78** linked to glycidoxypropyltrimethoxysilane (GPTMS)-modified TiO_2_ nanoparticles (Scheme 30). The corrole-GPTMS/TiO_2_ nanoparticles **79** demonstrated high photodegradation efficiency and reusability [71].

**Scheme 31:**
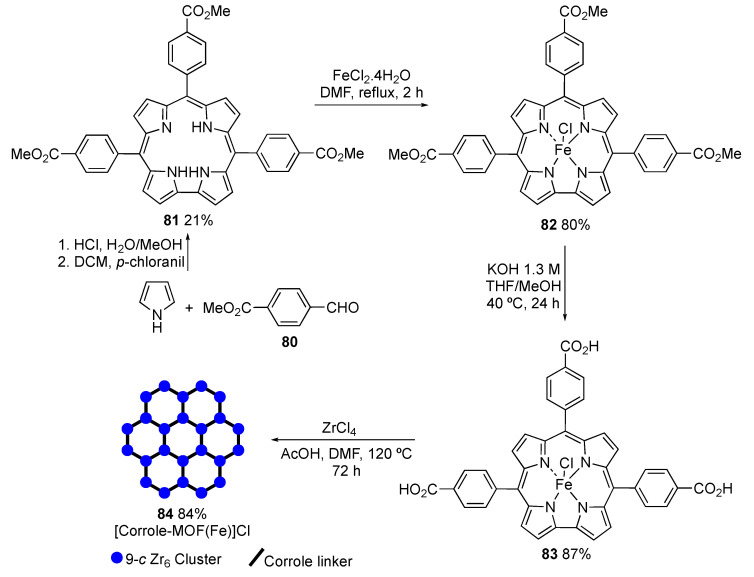
Synthesis of a heterogenous catalyst corrole-metal–organic framework (MOF).

Metal–organic frameworks (MOFs) are porous crystalline materials composed of a three-dimensional (3D) network of metal, held in place by multidentate organic molecules. Zhang and coworkers developed robust corrole-based MOFs (e.g., **84**) using 9-connected Zr_6_ or Hf_6_ and corrole as the organic linker (Scheme 31) [72]. The free base corrole **81** was prepared in 21% by the reaction of pyrrole with methyl *p*-formylbenzoate (**80**) in the presence of HCl, followed by oxidation with *p*-chloranil. The metalation with FeCl_2_ and hydrolysis of the ester group afforded the corrole (e.g., **83**) in good yield. Polycrystalline [corrole-MOF(Fe)Cl] **84** was isolated in 84% yield from the reaction of corrole **83** with ZrCl_4_ in DMF at 120 °C in the presence of acetic acid. This procedure was also applied to synthesize other corrole-MOFs, namely, using HfCl_4_ as metal source or the free base corrole as a linker. [Corrole-MOF(Fe)Cl] **84** was successfully used as a heterogeneous catalyst in hetero-Diels-Alder reactions of dienes with inactivated aldehydes.

**Scheme 32:**
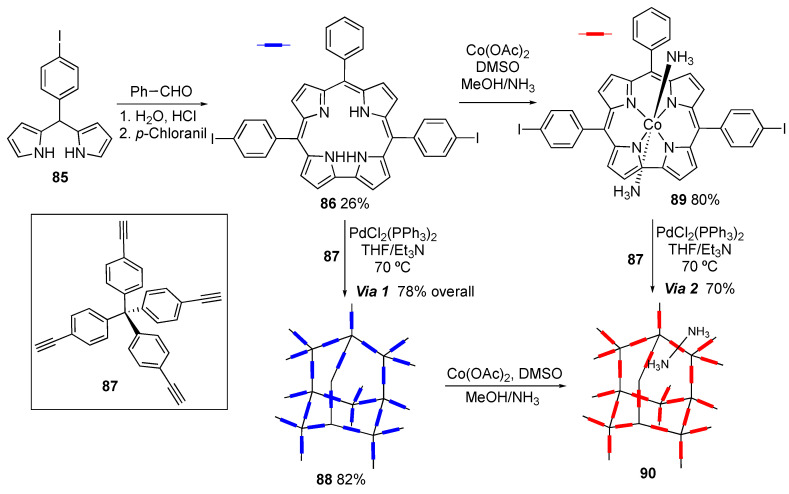
Synthetic routes to porous organic polymers (POP)-CorCo.

Many of the porous solids, such as MOFs, are not stable enough for some applications, due to their being built through coordination bonds between the organic ligand and the metal donors. Thus, to overcome some instability, Porous Organic Polymers (POPs) emerged as a new class of porous materials, which are built by covalent bonding between organic building blocks [73]. The synthesis of POP-CorCo **88** and **90** can be made following two strategies, having di-iodophenyl corrole **86** as the starting point (Scheme 32) [74]. Corrole **86** was prepared in 26% yield by the acid-catalyzed condensation of *meso*-(*p*-iodophenyl)dipyrromethane (**85**) with benzaldehyde, followed by oxidation with *p*-chloranil. The first strategy (via 1) to prepare POP-CorCo **88** is the reaction between the free base di-iodophenyl corrole **86** and the tetrahedral-shaped **87** through the copper-free palladium-catalyzed Sonogashira cross-coupling, followed by cobalt complexation, leading the target POP in 78% overall yield. The second methodology (via 2) involves the preparation of cobalt corrole complex **89**, protected by two amine ligands, followed by the coupling reaction to give the POP-CorCo **90** in 70% yield. The POP-CorCo **90** prepared by the two pathways exhibits similar high permanent porosity and the ability to selectively capture or sense carbon monoxide, even in the presence of other gases, such as CO_2_, O_2_ and N_2_.

## 5. Corroles as PDT Agents in Cancer Treatment

The chemical, physical and photophysical properties of corroles make them valuable photosensitizers for PDT. Therefore, the development of photosensitizers based on the corrole macrocycle has attracted the attention of several research groups and some review articles on this topic have been published [9,19,20,75]. Herein, the compilation of the latest advances is presented.

**Scheme 33:**
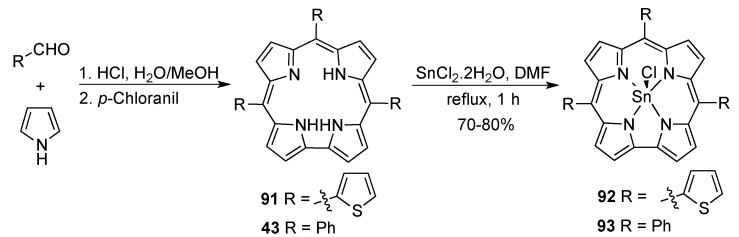
Synthesis of Sn(IV) *meso*-triarylcorroles.

Mack and coworkers described the synthesis and characterization of two Sn(IV) corroles with thien-2-yl (**92**) and phenyl (**93**) substituents at *meso*-positions and their photodynamic activity against breast cancer cells (MCF-7) [76]. The condensation of pyrrole with the appropriate aldehyde in presence of HCl followed by oxidation with *p*-chloranil gave corroles **91** and **43**. Sn(IV) triarylcorroles **92** and **93** were obtained via metalation with SnCl_2_ in refluxing DMF (Scheme 33). Sn(IV) tris-thyen-2-yl corrole **92** shows an absorption spectrum red shift compared with the phenyl analogue **93**, which leads to a better absorption in the therapeutic window. Along with the absence of aggregation in solution, tin(IV) complexes **92** and **93** have high values of singlet oxygen quantum yields (0.87 and 0.54, respectively) and triplet state lifetimes of 31 and 50 μs, respectively. The cellular uptake of both corrole complexes in MCF-7 cancer cells reached a peak after 24 h of incubation, with the concentration of **92**, two times higher than **93**. The photocytotoxicity assays of Sn(IV) corroles **92** and **93** show values of IC_50_ of 3.2 and 13.1 μM against MCF-7 cancer cells after irradiation and non-toxicity in the dark. The higher photocytotoxicity of corrole complex **92** was attributed to its high singlet oxygen quantum yield and lipophilicity.

**Scheme 34:**
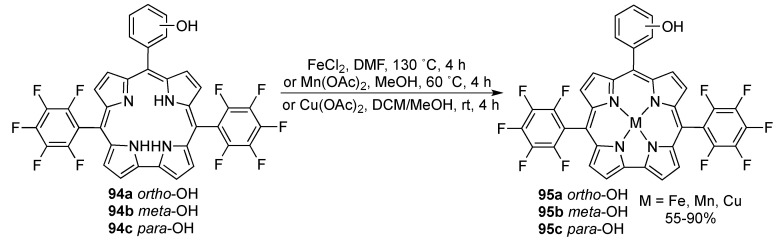
Synthesis of mono-hydroxy metallocorroles.

Mono-hydroxyphenyl metallocorroles **95a**–**c** have been prepared from the corresponding free base corroles **94a**–**c** by complexation with iron(III), manganese(III) and copper(III) in high yields (Scheme 34) and their DNA-binding ability and photodynamic antitumor activity were evaluated [77]. The DNA binding studies indicate that metallocorroles **94a**–**c** bind to CT-DNA by an external binding mode, with the *ortho*-hydroxyphenyl metallocorroles (**95a**) being the ones with less binding affinity. Cytotoxicity assays were performed using carcinoma A549 (lung), HepG2 (liver), MCF-7 (breast), DU145 (prostate) cell lines and one normal cell line, GES-1 (gastric epithelial). Under dark or light conditions, all the metallocorroles are non-toxic for the normal cells GES-1 and only **Cu**-**95b** was slightly cytotoxic against DU145 cells. On the other hand, manganese(III) and copper(III) corroles revealed weak cytotoxicity against A549, HepG2 and MCF-7 when compared with iron(III) corroles, in the absence or presence of light. In addition, **Fe**-**95c** shows a significant photocytotoxicity against A549 cells with IC_50_ of 30 μM, being noncytotoxic in the dark. The studies demonstrated that **Fe**-**95c** induced the A549 cell apoptosis and necrosis, high cellular ROS level and disruption of the mitochondrial membrane potential.

**Scheme 35:**
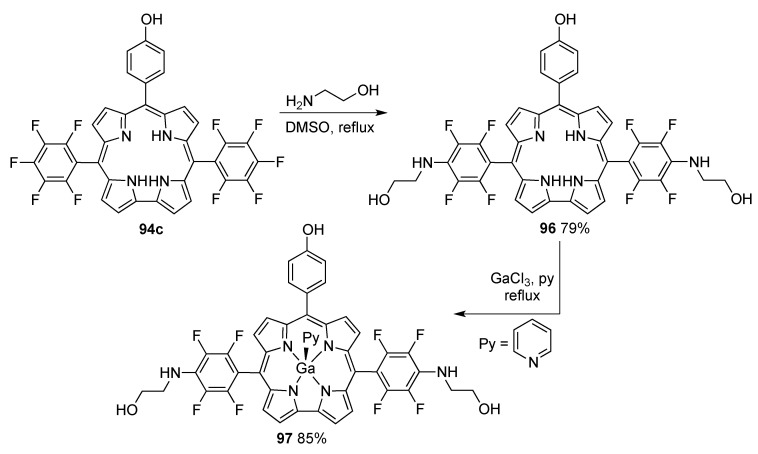
Synthesis of a tri-hydroxy corrole and the corresponding gallium complex.

Tri-hydroxy corrole **96** was prepared in high yield (79%) from corrole **94c** by aromatic nucleophilic substitution using 2-aminoethanol as the nucleophile, followed by complexation with gallium(III) to produce the metallocorrole **97** in 85% yield (Scheme 35) [78]. Similarly to mono-hydroxy complexes **95**, corroles **96** and **97** established outside binding with DNA. Corroles **96** and **97** showed moderate cytotoxicity in the dark, with corrole complex **97** being more cytotoxic than the free base **96**, and both exhibited high photocytotoxicity against lung cancer cells A549, hepatoma cell HepG2 and breast cancer cell MCF-7 (IC_50_ = 0.3 μM). The PDT treatment of HepG2 cells with **96** or **97** induces the potential destruction of the mitochondrial membrane and, consequently, tumor cell apoptosis.

**Figure 3:**
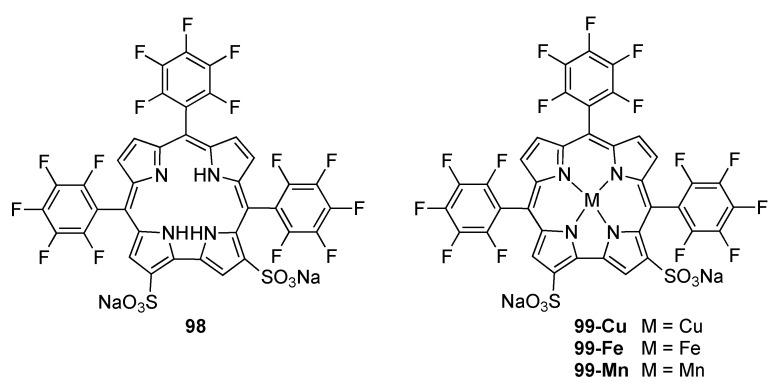
Molecular structure of a sodium salts of sulfonated corrole and its metal complexes.

Water-soluble sodium salts of sulfonated corrole **98** and its metal complexes of copper (**99**-**Cu**), iron (**99**-**Fe**) and manganese (**99**-**Mn**) were prepared and their photodynamic anti-cancer activity against human tumor cell lines, namely lung carcinoma (A549), hepatocellular cancer (HepG2) and cervical cancer (HeLa), was evaluated (Figure 3) [79]. Photocytotoxicity studies demonstrated that all the tested cell lines are sensitive to the presence of corrole **98**, especially A549 cells (IC_50_ = 5.0 μM), while no dark toxicity was observed with the studied corroles. Similar behavior was already found for corrole–cyclodextrin conjugates when compared with the corrole-free base **70** [80]. The studies to clarify the mechanism of PDT activity of corrole **98** evidenced a large production of reactive oxygen species (ROS), which could induce A549 cell apoptosis and activate the mitochondrial apoptosis, with the SIRT1 protein degradation being crucial for the process. The PDT activity of corrole **98** in vivo was also assessed and a significant decrease in tumor growth (A549 xenografted tumor), without obvious loss of mice body weight, was observed.

Re^V^O 5,10,15-tris(*meta*-methoxycarbonylphenyl)corrole, Re^V^O 5,10,15-tris(*para*-methoxycarbonylphenyl)corrole and their corresponding carboxylic acids were evaluated as sensitizers for PDT. This metallocorroles present a high singlet oxygen formation quantum yield (0.72) regardless of the ester or acid functionalities at the phenyl rings. The Re^V^O carboxylic acids were found to exhibit high photocytotoxicity against rat bladder cancer cells (AY27) and human colon carcinoma cells (WiDr), achieving 50% cell death within 5–7 min of blue light exposure. Cell death rates for WiDr cell line in the absence of light are much lower (23% cell death after 24 h incubation with a 10 μM concentration of sensitizer) [81].

**Scheme 36:**
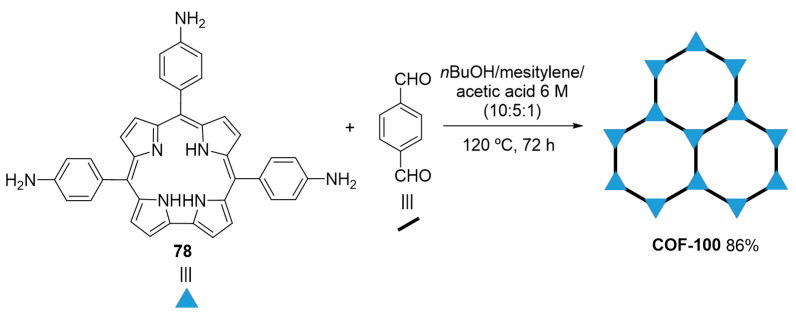
Synthesis a two-dimensional corrole-based covalent organic framework.

Recently, the synthesis of a two-dimensional corrole-based covalent organic framework (COF) was developed. The corrole-based **COF**-**100** with desymmetrized structure was isolated in 86% yield from the [3 + 2] imine condensation reaction of the approximately T-shaped tris(*p*-aminophenyl)corrole (**78**) with the linear terephthaldehyde (Scheme 36) [82]. **COF**-**100** demonstrated high chemical stability and permanent porosity, with a sphere-like morphology and a Brunauer–Emmett–Teller (BET) surface area of 745 m^2^/g. The uv-vis absorption spectrum shows an absorption intensity in the entire visible and NIR regions with a Soret band at 399 nm, which is redshifted by 7 nm relative to corrole **78**. **COF**-**100** exhibits a high capability to generate oxygen singlet (90% efficiency of 1,3-diphenylisobenzofuran (DPBF) degradation, whereas monomeric corrole showed sharply decreased activity for degrading DPBF with a low conversion of 56%), weaker fluorescence intensity and a shorter fluorescence lifetime of 0.082 ns compared with **78** (0.19 ns). Laser irradiation (0.18 W cm^−2^) of MCF-7 cells incubated with **COF**-**100** (50 μg/mL), at 635 nm for 10 min, led to the death of 85% of the cancer cells. These results clearly demonstrated that **COF**-**100** is a promising candidate to be used as a photosensitizer in photodynamic therapy. 

In summary, the efforts to develop new corrole-based entities useful as a photosensitizer for photodynamic therapy have followed different strategies: (i) optimization of the photophysical parameters through halogenation at *meso* or *beta* positions of the corrole core, where the iodination at 3-position of the *meso*-phenyl ring and beta-pyrrolic positions stand out, leading to the increase in singlet oxygen quantum yield; (ii) modification of the inner core through complexation with metals, namely Fe, Sn, Ir and Re complexes, which proved to be more efficient singlet oxygen sensitizers than the free-base corroles, with the Ga complexes also being more photocytotoxic; (iii) solubility issues have been overcome through the introduction of polar groups (SO_3_Na or COOH), by the formation of macrostructures such MOF or nanoparticles; (iv) the modification of uv-vis-NIR absorption characteristics was achieved by extending the π-conjugated system.

## 6. Hexaphyrins: Synthesis and Applications

Gossauer et al. described, in 1983, the first synthesis of hexaphyrins with six *meso*-bridges, β-substituted aromatic macrocycles with 26-electron conjugation, following a “3 + 3” approach, i.e., through the condensation of two tripyrromethanes [83]. Following this work, *meso*-substituted [28]hexaphyrins were synthetized using the classical methodologies for the synthesis of porphyrins, such as Lindsey and Rothemund synthetic approaches [84,85]. Although these methods are convenient and effective, when stabilized with electron-withdrawing groups at the *meso*-position, the simultaneous formation of every size of expanded porphyrins, at least up to dodecamer, inevitably causes serious separation difficulties. Therefore, more ring-size selective syntheses using dipyrromethanes and tripyrromethanes as the starting materials were developed [86,87].

**Scheme 37:**
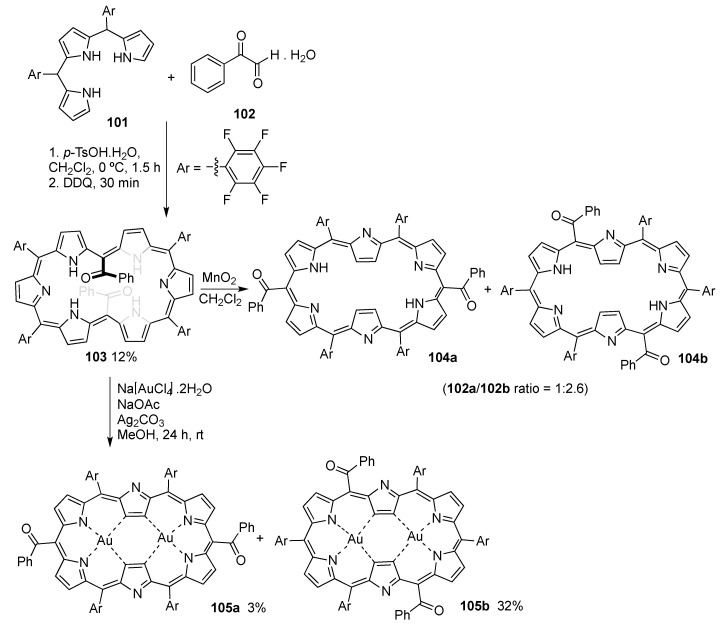
Synthesis of isomeric 5,20-dibenzoyl [28]hexaphyrins and their Gold(III) complexes.

More recently, Osuka and coworkers described the synthesis of hexaphyrins bearing two benzoyl groups at *meso*-positions and their complexes [88]. [28]Hexaphyrin **103** was synthesized as a stable compound in 12% overall yield by *p*-toluenesulfonic acid catalyzed cross-condensation of tripyrrane **101** with phenylglyoxal monohydrate (**102**), at 0 °C under N_2_ atmosphere with protection from room light for 1.5 h, followed by oxidation with DDQ (Scheme 37). X-Ray diffraction analysis has revealed that **103** adopts a dumbbell-like conformation, held by effective intramolecular hydrogen bonding between the pyrrolic NH protons and benzoyl carbonyl groups. Oxidation of **103** with MnO_2_ afforded [26]hexaphyrin **104**, quantitatively. However, hexaphyrin **104** is easily reduced back to **103** under ambient conditions, indicating that [28]hexaphyrin is more stable than [26]hexaphyrin despite its distinct antiaromatic character. The complexation of **103** with Au(III) afforded two bis-Au(III) isomeric complexes **105a** and **105b** in 32% and 3%, respectively (Scheme 37). X-Ray crystal analysis showed that both complexes present rectangular conformations with hydrogen bonding between the benzoyl groups and the outer NH protons. 

**Scheme 38:**
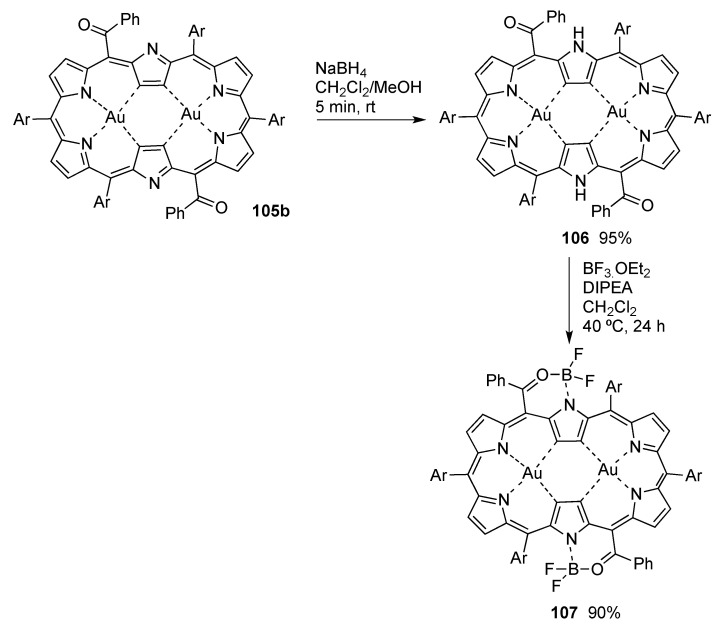
Synthesis of a [26]hexaphyrin bis-gold(III) complex and the corresponding exocyclic boron complex.

Complex **105b** was quantitatively reduced to [26]hexaphyrin Au(II) complex **106** with NaBH_4_ at room temperature, which could oxidize back to **105b** without significant degradation by treatment with MnO_2_. The benzoyl substituents and pyrrolic NH-protons of the bis-Au(III) complex of [28]hexaphyrin **106** were used as peripheral bidentate coordination sites to prepare the boron(II) complex **107**. The complexation was achieved in 90% yield by heating a mixture of **106**, BF_3_-OEt_2_ and DIPEA in dichloromethane at 40 °C for 24 h (Scheme 38) [88].

The absorption spectrum of the [28]hexaphyrin **103** in CH_2_Cl_2_ shows a Soret band at 516 nm and a Q band (without fine structure) between 700–900 nm. The absorption spectra of gold complexes **105a** and **105b** are similar, showing Soret-like bands around 670 nm and Q-like bands in the NIR region at ca. 822, 930 and 1195 nm, supporting their 26π aromaticity. The absorption spectrum of the reduced complex **106** shows broad absorption bands at 544, 603, and 738 nm and weak absorption in NIR region, reflecting its antiaromaticity. The absorption spectrum of **107** is red-shifted by ca. 100 nm and sharper than that of **106**, probably due to structural rigidity and the presence of electron withdrawing BF_2_-moieties.

**Scheme 39:**
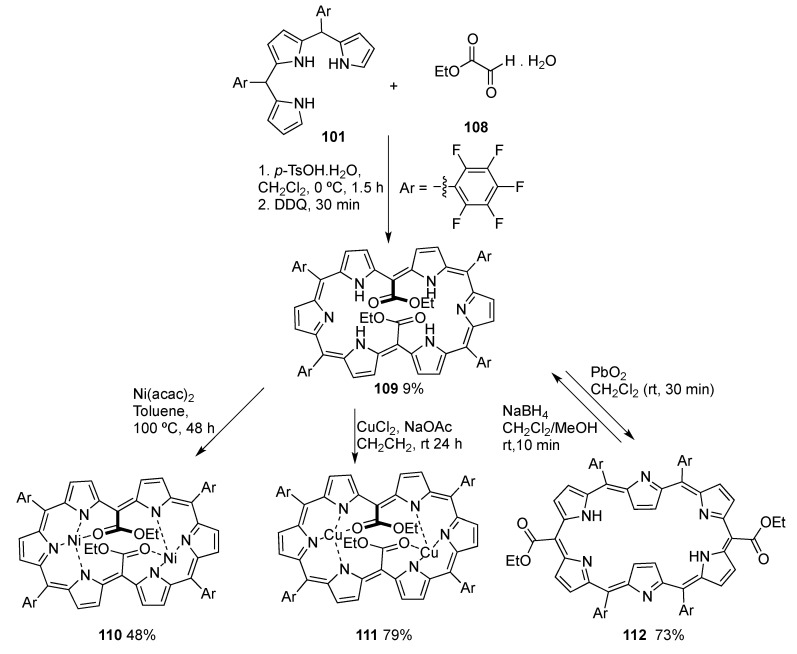
Synthesis of a 5,20-bis(ethoxycarbonyl)-[28]hexaphyrin, its bis-Ni(II) and bis-Cu(II) complexes and the corresponding 5,20-bis(ethoxycarbonyl)-[26]hexaphyrin.

5,20-Bis(ethoxycarbonyl)-[28]hexaphyrin **109** was synthesized using *meso*-pentafluorophenyl tripyrrane **101** and ethyl 2-oxoacetate (**108**) as starting materials and the previously described methodology to promote the cross-condensation, followed by oxidation with DDQ (Scheme 39) [89]. X-Ray crystallographic analysis revealed that **109** presents a dumbbell-like conformation held by effective intramolecular hydrogen bonding between the pyrrolic NH protons and carbonyl oxygen of ethoxycarbonyl group. The uv-vis spectrum of **109** displayed characteristic features of antiaromatic porphyrinoids with a broad Soret-like band at 514 nm, and relatively weak Q-like bands. [28]Hexaphyrin **109** can serve as a bis-NNNO ligand to form square planar bis-Ni(II) and bis-Cu(II) complexes. The reaction of **109** with 20 equiv. of nickel(II) acetylacetonate (Ni(acac)_2_) in dry toluene at 100 °C for 48 h afforded a bis-Ni(II) complex **110** in 48% yield. X-Ray crystallographic analysis has confirmed that **110** has a square planar geometry formed through the coordination of the three nitrogen atoms inner and the carbonyl oxygen atom to the Ni(II) ion, and that this bis-complex kept the dumbbell-like conformation. The complexation with a large excess of CuCl_2_ in the presence of NaOAc in CH_2_Cl_2,_ at room temperature, for 24 h, yields the bis-Cu(II) complex **111** in 79% yield, which also takes on a dumbbell-like conformation and square planar geometry. The absorption spectrum of **110** in CH_2_Cl_2_ showed a broad Soret-like band at 508 and 621 nm, Q-like band at 830 nm and a weak absorption tail up to 1700 nm. The complexation with copper modified the absorption spectrum of **111**, narrowing the Soret-like band at 520 and 635 nm, blue-shifting the Q-like bands to 814 nm and also the very weak absorption tail up to ca. 1500 nm. Oxidation of 5,20-bis(ethoxycarbonyl)-[28]hexaphyrin **109** with a large excess of PbO_2_ in CH_2_Cl_2_ at room temperature for 3 min afforded [26]hexaphyrin **112** in 73% yield. This hexaphyrin was easily reduced back to **109** with NaBH_4_ in a mixture of CH_2_Cl_2_/MeOH at room temperature for 10 min (Scheme 39). Interestingly, the hexaphyrin **112** was the sole product, in contrast with the formation of two isomeric [26]hexaphyrins in the oxidation of 5,20-dibenzoyl-[28]hexaphyrin with MnO_2_ [88]. The uv-vis-NIR absorption spectrum of **112** showed a sharp Soret-like band at 562 nm and Q-like bands at 712, 770, 892, and 1022 nm, characteristic features of aromatic porphyrinoids.

**Scheme 40:**
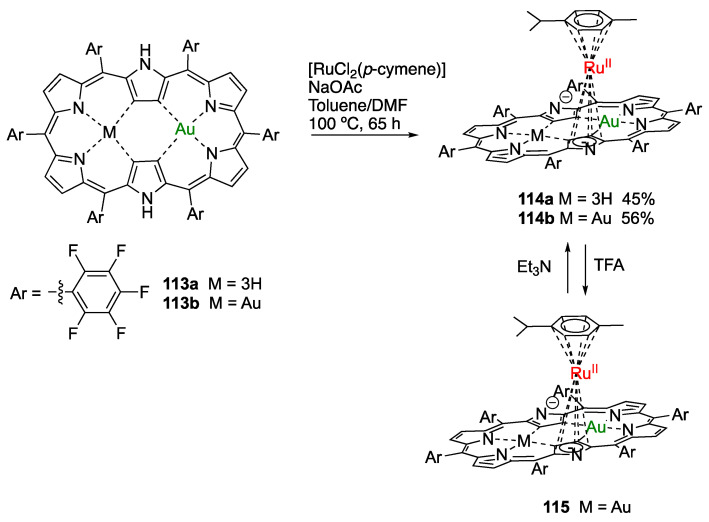
Synthesis of Ru(II) complexes of *meso*-pentafluorophenyl-[28]hexaphyrin mono- and bis-Au(III) complexes.

Following studies on the complexation of aromatic electron-deficient *meso*-pentafluorophenyl [26]hexaphyrin and electron rich *meso*-pentafluorophenyl [28]hexaphyrin with Möbius aromaticity, Osuka and coworkers reported the synthesis of π-ruthenium complexes [90]. The treatment of *mono*- and bis-gold(III) complexes of [28]hexaphyrin **113a** and **113b** with five equivalents of [RuCl_2_(*p*-cymene)] in the presence of NaOAc in a mixture of toluene/DMF at 100 °C for 65 h afforded π-ruthenium complexes **114a** and **114b** in 45% and 56% yield, respectively (Scheme 40). When the *meso*-pentafluorophenyl [26]hexaphyrin was submitted to the same reaction conditions, only decomposition products were obtained, suggesting that the formation of ruthenium π-complexes might need an electron-rich hexaphyrin. The absorption spectrum of **114b** shows two intense bands at 490 and 656 nm and two less intense and more broad bands at 1066 and 1250 nm. The treatment with TFA yielded the trifluoroacetate **115**, with the absorption spectrum of this salt presenting the same bands, and blue shifted to 455, 609, 1066 and 1097 nm. 

**Scheme 41:**
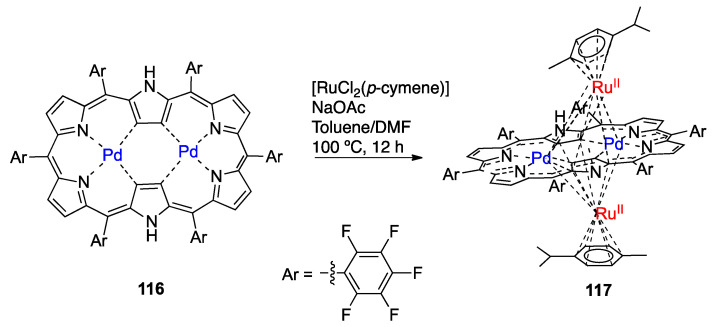
Synthesis of Ru(II) complexes of *meso*-pentafluorophenyl-[28]hexaphyrin bis-Pd(II) complex.

Ru-metalation of [26]hexaphyrin bis-Pd(II) complex **116**, a unique complex with a characteristic conjugated aromatic circuit and relatively acidic outer pyrrolic NH protons [27,91], was achieved through a reaction with [RuCl_2_(*p*-cymene)] in the presence of NaOAc in a mixture of toluene/DMF (4:1) at 100 °C for 12 h [90]. The [26]hexaphyrin bis-Pd(II)-bis-Ru(II) complex **117**, a triple-decker complex with both the [(*p*-cymene)Ru(II)] moieties located above and below the center of the hexaphyrin framework, was obtained in 57% yield (Scheme 41). The absorption spectrum of **117** in CH_2_Cl_2_ shows two Soret-like bands at 461 and 650 nm and a Q-like band at 829 nm.

**Scheme 42:**
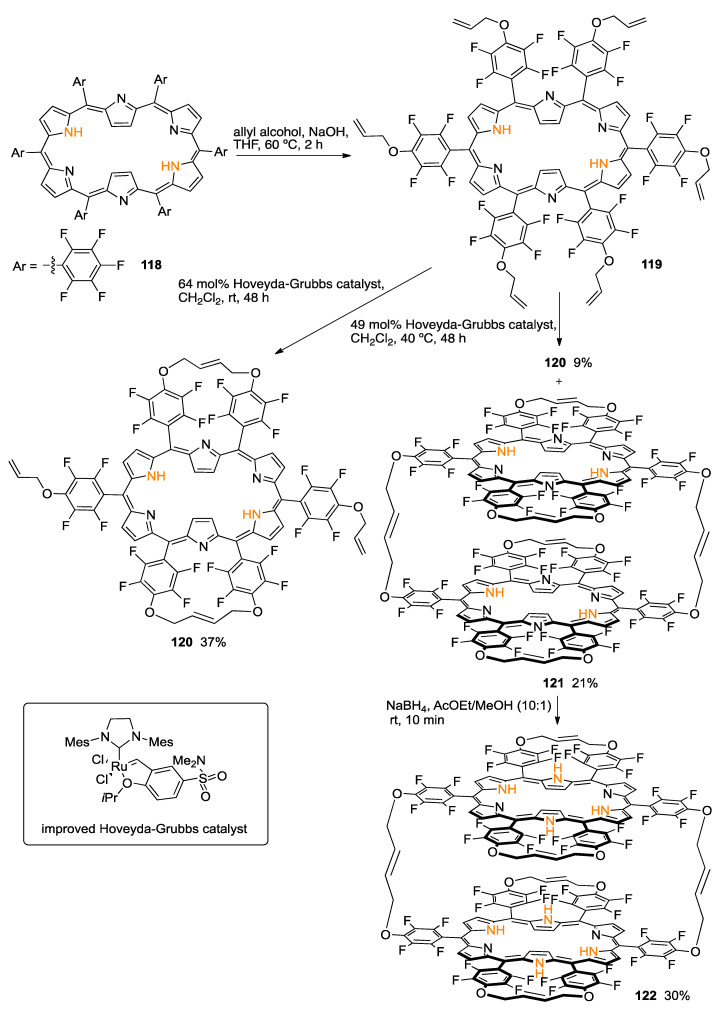
Synthesis of monomeric and dimeric strapped hexaphyrins.

Hexaallyloxy-appended hexaphyrin **119** prepared through nucleophilic aromatic substitution reaction of 5,10,15,20,25,30-*hexakis*(pentafluorophenyl)-[26]hexaphyrin (**118**) with allyl alcohol, [92] was subjected to olefin metathesis reaction using the improved Hoveyda–Grubbs catalyst. When the reaction was conducted at room temperature, only peripherally strapped [26]hexaphyrin monomer **120** was obtained in a 37% yield. Decreasing the amount of catalyst and increasing the reaction temperature to 40 °C led to the formation of **120** as a minor product (9% yield) and cyclophane-type [26]hexaphyrin dimer **121** in 21% yield (Scheme 42). The treatment of dimer **121** with NaBH_4_ at room temperature for 10 min afforded [28]hexaphyrin dimer **122** in 30% yield, which could be transformed back into **121** by oxidation with MnO_2_ (Scheme 42) [93]. The uv-vis-NIR absorption spectrum of aromatic [26]hexaphyrin **120** shows a typical sharp Soret-like band at 569 nm and Q-like bands at 718, 908, and 1031 nm. Dimer **121** shows split Soret-like bands at 543 and 577 nm, attributed to the exciton coupling of the two hexaphyrin cores in a stacked geometry, [94] and Q-like bands 724, 899, and 1031 nm. The absorption spectrum of **122** exhibits characteristics of anti-aromatic [28]hexaphyrins, smaller absorption bands at 490, 526, and 571 nm and a weak absorption at the near-infrared region.

**Scheme 43:**
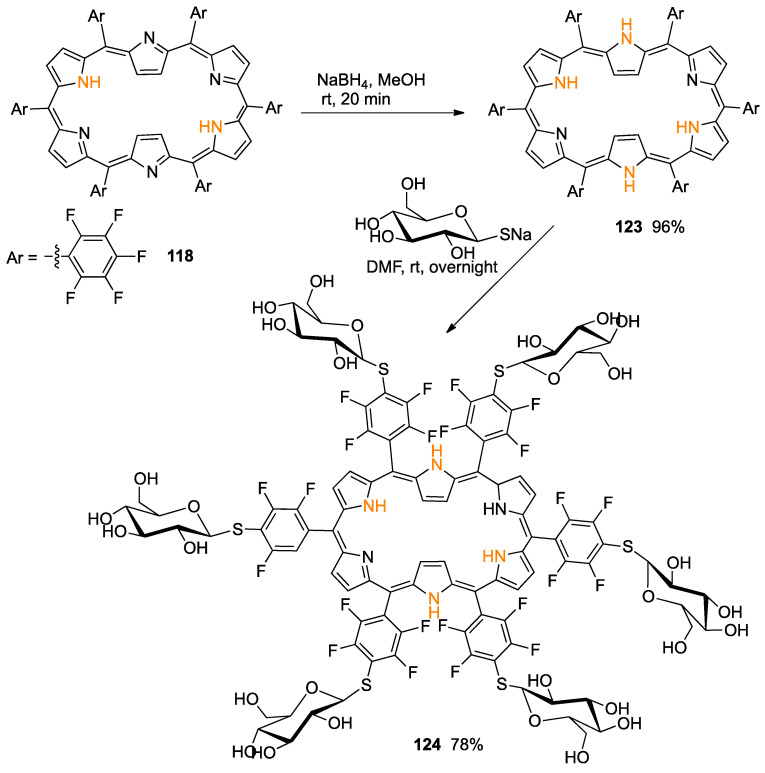
Synthesis of a hexa-glycosylated [28]hexaphyrin.

Following the pioneer work of Osuka and coworkers on regioselective nucleophilic substitution reactions of *meso*-*hexakis*(pentafluorophenyl)-substituted [26]hexaphyrin with an alkoxides and isopropyl amine [95], Wiehe and coworkers achieved the synthesis of hexa-glycosylated [28]hexaphyrin **124** in high yield (Scheme 43) [96]. [28]Hexaphyrin **123**, obtained in 96% yield through a reduction in [26]hexaphyrin **118** with NaBH_4_ in methanol at room temperature for 20 min, was transformed into the hexa-glycosilated derivative in 78% yield by nucleophilic substitution of the *para* fluorine substituent of all six pentafluorophenyl units, using 1-thio-β-D-glucose sodium salt as nucleophile, in dry DMF overnight at room temperature. Attempts to promote the glycosylation of [26]hexaphyrin **118** delivered a complex mixture with traces of the expected product and evidence for the formation of the corresponding reduced form, [28]hexaphyrin **123**.

**Figure 4:**
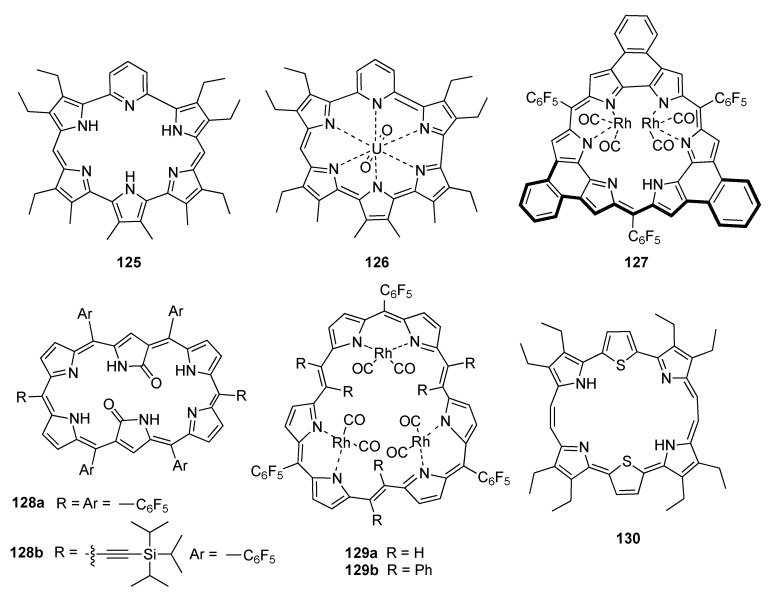
Other hexaphyrins derivatives and analogs synthesized in recent years.

To create well-defined NIR-II dyes, chemical modification of the hexapyrrolic core is an alternative viable approach. Recently, Sessler and coworkers reported the synthesis of a new hexaphyrin, pyrihexaphyrin (0.1.0.0.1.0) **125**, (Figure 4) that, upon uranyl dication complexation, undergoes ring contraction, affording 22 π-electron aromatic contracted pyrihexaphyrin (0.0.0.0.1.0)-uranyl complex, **126**, which displays Hückel type aromatic features. In fact, uv-vis spectroscopic analysis revealed marked differences in comparison to the precursor. A relatively weak Soret-like band was observed at 549 nm along with Q-type bands at 734 and 1003 nm [97]. A robust bis-rhodium(I) complex of π-extended planar, anti-aromatic *meso*-pentafluorophenyl-β,β-phenylene-bridged hexaphyrin [1.0.1.0.1.0], rosarin **127**, was synthesized in 60% yield from the corresponding free-base (Figure 4). Both the ligand and the bis-Rh(I) complex show uv-vis spectra in CH_2_Cl_2_ with broad bands between 400–700 nm [98].

The synthesis of doubly *N*-confused dioxohexaphyrins **128a**,**b** as well as their Zn and Cu complexes, was also reported (Figure 4). These are interesting small molecule-based photoacoustic agents that respond to NIR-II light excitation and constitute a promising platform for deep-tissue NIR-II photoacoustic imaging applications [99].

Hexaphyrin (2.1.2.1.2.1), also known as rubyrins, **129a** and **b** and their rhodium, zinc and copper metal complexes, were obtained to explore the control of aromaticity and *cis*-/*trans*-isomeric structure of non-planar hexaphyrins (Figure 4). While the free-base and Zn complex exhibit uv-vis spectra similar to the ones observed for porphyrins, the copper complex has a significant red-shifted bands with Q-like bands at 859 and 947 nm. The rhodium complex displayed a sharp, intense Soret-like band, and a weak Q-like band in the NIR region [100,101].

The incorporation of chalcogenic elements into five-membered rings (i.e., furan and thiophene) significantly affects the dipole vector and charge displacement. This causes a reversal in the direction of the dipole moment between the furan/thiophene and pyrrole [102]. The change in the polarization results in electronic perturbations of the π conjugation. In this regard, chalcogen-substituted hexaphyrins have been synthesized [103,104,105,106,107,108], some of them having been explored as NIR photodynamic therapy agents [109], and others such as dithiabronzaphyrin **130** [108] show intense absorption and fluorescence in the NIR region, opening the way for their use in biological applications.

## 7. Conclusions

The efforts of several research groups to develop the synthesis of corroles and hexaphyrins followed three main strategies: (a) improvement in classical methodologies for the synthesis of the macrocycle; (b) development of methodologies for the functionalization of the peripheral positions and (c) improvement in complexation methods to modify the inner core. In addition to the above, a few novel methodologies have been developed and all, together, opened the way to corroles and hexaphyrins with new substitution patterns. The resulting modulation of the photophysical properties lays the foundation for the development of further applications.
